# Calcium intake and genetic variants in the calcium sensing receptor in relation to colorectal cancer mortality: an international consortium study of 18,952 patients

**DOI:** 10.1038/s44276-024-00077-3

**Published:** 2024-09-02

**Authors:** Evertine Wesselink, William Gauderman, Sonja I. Berndt, Hermann Brenner, Daniel D. Buchanan, Peter T. Campbell, Andrew T. Chan, Jenny Chang-Claude, Michelle Cotterchoi, Marc J. Gunter, Michael Hoffmeister, Amit D. Joshi, Christina C. Newton, Rish K. Pai, Andrew J. Pellatt, Amanda I. Phipps, Mingyang Song, Caroline Y. Um, Bethany van Guelpen, Emily White, Ulrike Peters, Fränzel J. B. van Duijnhoven

**Affiliations:** 1 Division of Human Nutrition and Health, Wageningen University & Research, Wageningen, The Netherlands; 2 Division of Biostatistics, Department of Population and Public Health Sciences, Keck School of Medicine, University of Southern California, Los Angeles, CA USA; 3 Division of Cancer Epidemiology and Genetics, National Cancer Institute, National Institutes of Health, Bethesda, MD USA; 4 Division of Clinical Epidemiology and Aging Research, German Cancer Research Center (DKFZ), Heidelberg, Germany; 5 Division of Preventive Oncology, German Cancer Research Center (DKFZ) and National Center for Tumor Diseases (NCT), Heidelberg, Germany; 6 German Cancer Consortium (DKTK), German Cancer Research Center (DKFZ), Heidelberg, Germany; 7 Colorectal Oncogenomics Group, Department of Clinical Pathology, Melbourne Medical School, The University of Melbourne, Parkville, VIC Australia; 8 University of Melbourne Centre for Cancer Research, The University of Melbourne, Parkville, VIC Australia; 9 Genomic Medicine and Family Cancer Clinic, The Royal Melbourne Hospital, Parkville, VIC Australia; 10 Department of Epidemiology and Population Health, Albert Einstein College of Medicine, Bronx, NY USA; 11 Division of Gastroenterology, Massachusetts General Hospital and Harvard Medical School, Boston, MA USA; 12 Channing Division of Network Medicine, Brigham and Women’s Hospital and Harvard Medical School, Boston, MA USA; 13 Clinical and Translational Epidemiology Unit, Massachusetts General Hospital and Harvard Medical School, Boston, MA USA; 14 Broad Institute of Harvard and MIT, Cambridge, MA USA; 15 Department of Epidemiology, Harvard T.H. Chan School of Public Health, Harvard University, Boston, MA USA; 16 Division of Cancer Epidemiology, German Cancer Research Center (DKFZ), Heidelberg, Germany; 17 University Medical Centre Hamburg-Eppendorf, University Cancer Centre Hamburg (UCCH), Hamburg, Germany; 18 Prevention and Cancer Control, Cancer Care Ontario, Toronto, ON Canada; 19 Dalla Lana School of Public Health, University of Toronto, Toronto, ON Canada; 20 Nutrition and Metabolism Branch, International Agency for Research on Cancer, World Health Organization, Lyon, France; 21 Department of Epidemiology and Biostatistics School of Public Health Imperial College London, London, UK; 22 Department of Population Science, American Cancer Society, Atlanta, Georgia USA; 23 Department of Laboratory Medicine and Pathology, Mayo Clinic Arizona, Scottsdale, AZ USA; 24 Department of Cancer Medicine, University of Texas MD Anderson Cancer Center, Houston, TX USA; 25 Department of Epidemiology, University of Washington, Seattle, WA USA; 26 Public Health Sciences Division, Fred Hutchinson Cancer Center, Seattle, WA USA; 27 Departments of Epidemiology and Nutrition, Harvard T.H. Chan School of Public Health, Harvard University, Boston, MA USA; 28 Department of Radiation Sciences, Oncology Unit, Umeå University, Umeå, Sweden; 29 Wallenberg Centre for Molecular Medicine, Umeå University, Umeå, Sweden

## Abstract

**Background:**

Research on calcium intake as well as variants in the calcium sensor receptor (*CaSR)* gene and their interaction in relation to CRC survival is still limited.

**Methods:**

Data from 18,952 CRC patients, were included. Associations between primarily pre-diagnostic dietary (*n* = 13.085), supplemental (*n* = 11,837), total calcium intake (*n* = 5970) as well as 325 single nucleotide polymorphisms (SNPs) of the *CaSR* gene (*n* = 15,734) in relation to CRC-specific and all-cause mortality were assessed using Cox proportional hazard models. Also interactions between calcium intake and variants in the *CaSR* gene were assessed.

**Results:**

During a median follow-up of 4.8 years (IQR 2.4–8.4), 6801 deaths occurred, of which 4194 related to CRC. For all-cause mortality, no associations were observed for the highest compared to the lowest sex- and study-specific quartile of dietary (HR 1.00, 95%CI 0.92–1.09), supplemental (HR 0.97, 95%CI 0.89–1.06) and total calcium intake (HR 0.99, 95%CI 0.88–1.11). No associations with CRC-specific mortality were observed either. Interactions were observed between supplemental calcium intake and several SNPs of the *CaSR* gene.

**Conclusion:**

Calcium intake was not associated with all-cause or CRC-specific mortality in CRC patients. The association between supplemental calcium intake and all-cause and CRC-specific mortality may be modified by genetic variants in the *CaSR* gene.

## Introduction

Epidemiologic studies provide considerable evidence for a protective association between calcium intake and the risk of colorectal cancer (CRC) [1–4]. In a dose-response meta-analyses including 15 studies and 12,305 CRC patients, each 300 mg/day increase in total calcium intake was associated with an approximately 8% reduced risk of CRC (RR 0.92 95%CI 0.89–0.95) [1]. A similar association was observed for dietary calcium intake (RR 0.90 95%CI 0.85–0.96) as well as supplementary calcium intake (RR 0.91 95%CI 0.86–0.98) [1].

In contrast to CRC risk [5], limited evidence is available for the association between calcium intake and survival in persons already diagnosed with CRC. The association between calcium intake and survival in CRC patients was examined in a total of 6 observational studies, involving between 148 and 3859 CRC survivors, with conflicting results [6–11]. No associations were observed for pre-diagnostic calcium intake in relation to all-cause and CRC-specific mortality in CRC patients [6–9]. An inverse association of post-diagnostic calcium intake with all-cause and CRC-specific mortality was observed in three cohort studies [6, 8, 10], but this was only statistically significant in one study for all-cause mortality [6] and in another study for CRC-specific mortality [8]. Thus, the relationship between calcium intake and mortality in CRC patients remains inconclusive.

Moreover, the underlying mechanisms by which calcium exerts its potential effect on CRC outcomes are still unknown. The inverse association between calcium intake and CRC risk is suggested to be mediated by the calcium-sensing receptor (CaSR) [12, 13], which is primarily activated by extracellular calcium. The CaSR plays a critical role in sensing of extracellular calcium to maintain serum calcium concentrations in a narrow physiological range. In the intestine, the CaSR is responsible for calcium absorption from the diet. Besides its primary function in the control of calcium homeostasis, the CaSR also has tumor suppressor functions as it can regulate inflammation, cell proliferation, cell differentiation and apoptosis [14, 15]. A lower expression of CaSR is associated with more aggressive tumors [15]. In addition, a higher expression of the CaSR in CRC tumor tissue was associated with a decreased CRC-specific mortality, but not all-cause mortality [14]. In addition, some indications for a gene-environment interaction between calcium intake and genetic variance of the *CaSR* gene were observed in CRC patients [16], where a specific haplotype of the *CaSR* gene seems to be associated with a decreased overall survival only in patients with a dietary calcium intake below the median. However, research on genetic variants of the *CaSR* gene and on the interaction between calcium intake and genetic variants in relation to CRC mortality is still limited.

Therefore, in this analysis, we examined the hypothesis that dietary and supplemental calcium intake is associated with all-cause and CRC-specific mortality with possible effect modification by genetic variants in the *CaSR* gene in a large population of 18,952 CRC patients.

## Methods

### Study design and participants

The study population for analyses consists of participants of studies included in the International Survival Analyses in Colorectal Cancer Consortium (ISACC), which is part of the Genetics and Epidemiology of Colorectal Cancer Consortium (GECCO). Data from 9 observational cohort studies, 3 clinical trials with a long-term follow-up and 2 case-control studies was used. Characteristics of the studies are listed in Table S1 and described in detail elsewhere [17–38].

In brief, for this study the participants from the mentioned studies who developed CRC during follow-up or were cases in the two case-control studies were selected. Only those with available dietary or supplemental calcium intake data were included for data-analyses. All participants gave written informed consent and studies were approved by the Institutional Review Boards.

#### Study population

In Table S1, the characteristics of the 14 included studies are summarized. Studies are conducted in the USA, Europe and Australia. The number of CRC patients included per study varied widely between 280 and 3654. Thirteen out of fourteen studies had data about dietary calcium intake available. Nine studies had data about supplemental intake available and eight studies had data about both dietary and supplemental intake available. All studies had data about all-cause mortality available, while thirteen studied had data about CRC-specific mortality available.

### Epidemiological data collection

Data about demographics, lifestyle and clinical factors was collected by self-report using structured questionnaires or in-person interviews. Information about how data was collected in each study can be found in Table S1.

Data from each included study was harmonized for ISACC. The methods of data-harmonization are described in detail elsewhere [20]. Data about study characteristics including country in which the study was conducted, study acronym and methods of exposure and outcome assessments was harmonized. In addition, information about the study population was harmonized: race, education level, sex, age at diagnosis, CRC stage, body mass index (BMI), physical activity level, dietary intake, calcium supplement use, follow-up time and clinical outcomes. Data on clinical outcomes were collected via regular follow up with confirmation using medical chart review, and or linkage with death and cancer registries (Table S1).

### Calcium intake assessment

Dietary intake, including dietary calcium intake, was measured using a food frequency questionnaire or diet history questionnaire in all studies. Dietary intake was measured before diagnosis (*n* = 14,792; median 3 years IQR 1–7), around diagnosis i.e., in the same year as the cancer diagnosis (*n* = 4153) or after diagnosis (*n* = 6). Length of the dietary questionnaires ranged from very short (19 items) to extended (178 items) (Table S1). Sex- and study- specific quartiles of calcium intake were used for analysis, because absolute values between studies may differ due to differences in the dietary assessment methods. Calcium from supplements (including single, multivitamins, and antacids) was measured in tablets per day. When actual quantities were unavailable, it was assumed that regular use of supplements was 500 mg/day or 500 mg/tablet for single calcium and antacids, and 130 mg/day or 130 mg/tablet for multivitamins. For the analyses, supplemental intake was defined as <1 pill (<500 mg) and ≥1 pill (≥500 mg). Total calcium intake was calculated for persons with both dietary and supplemental calcium intake data available and defined as sex- and cohort specific quartiles.

### Genotyping and SNP selection

From the included populations, blood samples have been sent for genotyping. In total, 15,734 blood samples could be successfully genotyped. Details on genotyping and quality control have been previously published [39] and genotyping platforms used are summarized in Table S1. DNA samples were validated with quality controls, and genotypic data that passed initial control were analysed by the analysis team of University of Washington Genetic Analysis Center. A call rate of >95% was applied and individuals from whom more than 95% of the typed SNPs was missing, were excluded. All SNPs of all studies were imputed to the Haplotype Reference Consortium r1.1 (2016) reference panel via the Michigan Imputation Server [40]. A candidate gene approach was used to investigate the interaction between calcium intake and genetic variance in the Calcium-Sensing Receptor (CaSR). The molecular location of the CaSR gene is base pairs 121,902,530–122,005,342 on chromosome 3 (GRCh37). In total, 1412 SNPs located in the *CaSR* region were selected for further analysis. After exclusion of 1087 SNPs as a result of MAF < 0.05, 325 SNPs were retained in the analysis (Table S3). All SNPs had an imputation accuracy of R^2^ > 0.85. For the genetic data analyses part, all participants were of Caucasian ancestry.

### Data analyses

Patient characteristics were described as medians with interquartile range (IQR) for the total population and by high versus low calcium intake (quartile 1 and 2 versus 3 and 4 of sex- and study-specific quartiles of intake). In addition, patients’ characteristics were described for each individual study.

The association between calcium intake (sex- and study- specific quartiles) and all-cause as well as CRC-specific mortality was assessed using two methods. First, a one stage model was applied, where individual data of participants of all studies were harmonized. The association was investigated using a Cox proportional hazards regression model.

Age, sex and cohort were included in the models *a priori*. Additionally, other potential confounders (education, family history of CRC, BMI, intake of total energy intake, folate, red meat, processed meat, fiber, vegetable, fruit and alcohol, physical activity, smoking status, regular aspirin/NSAID use, diabetes, and cancer site) were tested and included in the model when the HR changed by more than 10%. None of the mentioned potential confounders did change the HR with >10% and therefore only age, sex and cohort were included in the final models. To future explore timing of calcium intake, time between assessment of calcium intake and diagnosis in years was added to the model.

Subgroup analyses were done for sex (male, female), tumor location (proximal, distal, rectum), stage of disease (local, regional, distant), age at diagnosis (Early onset ≤50 years, late onset >50 years), family history of CRC (no, yes), timing of calcium intake (before diagnosis, around diagnosis) and study design (cohort, trial with follow-up, case-control), since associations between calcium intake and mortality could potentially be different for before mentioned subgroups [2, 3, 6, 8]. In a sensitivity analysis, data from two studies (DACHs and PHS) with very low calcium intake (median <440 mg/day), which was probably due to the restricted dietary assessment method, was excluded.

As a secondary analysis, a meta-analysis was conducted, where associations between calcium intake and mortality were first assessed for each study separately using Cox proportional hazards analyses. Models were adjusted for age and sex. Subsequently, obtained statistics were used to calculate a weighted average over all included studies. The DerSimonian and Laird (DL) random-effects model was used to account for heterogeneity of study populations and designs [41, 42]. The heterogeneity among the included studies was investigated using the I^2^ index and Cochran’s Q test, with significant heterogeneity assumed for I^2^ > 50% or a Q-test *p* < 0.05. Forest plots were made to visualize the data.

The associations between SNPs in the *CaSR* gene and mortality were assessed, assuming an additive model in which SNPs were encoded as 0,1,2, by using Cox proportional hazards regression analyses. In addition, SNPs were entered categorical as three groups (i.e., AA, Aa, aa). Models were adjusted for age, sex, study center and the first 3 principal components of genetic ancestry. These associations were investigated using a harmonized dataset of individual participant data.

Interaction between calcium and genetic variants in the *CaSR* gene was also investigated. For the interaction analyses only the additive model in which SNPs were encoded as 0,1,2 was used. We tested multiplicative interaction using SNP x calcium product terms, adjusting for age, sex, study center, first 3 principal components of genetic ancestry, and SNP and calcium main effects. Additive interaction was assessed by calculating the relative excess risk due to interaction (RERI) based on the estimates extracted from the multiplicative model (e^((β^Calcium^ + β^SNP^+β^Calcium*SNP^))-e^(β^Calcium^)-e^(β^SNP^) + 1). The delta method was used to estimate the variance and 95% confidence intervals (CI) of RERI [43]. A RERI of zero means no additive interaction, a RERI < 0 a negative additive interaction and a RERI > 0 a positive additive interaction. Sex- and study- specific quartiles as well as SNPs were entered as continuous variables for both the multiplicative as well as the additive interaction models.

To provide more insights into the nature of multiplicative and additive interactions between calcium intake and genetic variants in the *CaSR* gene, the 2 SNPs for which a multiplicative as well as an additive interaction for all-cause or CRC-specific mortality was observed were further investigated by A: joint effects of genotype (3 categories) and calcium intake (2 categories, median-split), where the reference group was a low calcium intake and the presence of the homozygous reference allele; and B: stratified analyses, examining the association of the SNP in relation to mortality in strata of calcium intake and the association of calcium intake in relation to mortality in strata of SNP genotypes.

All analyses were performed using R statistical software, version 4.0.3. The simple M method was used to calculate the number of independent tests for 325 SNPs [44]. To provide the number of effective test, additive SNP coding was used (0,1,2). The number of independent tests was 35, meaning that a *p*-value of 0.05/35 < 0.001 was considered statistically significant. Correlations between SNPs, SNPs in LD, were assessed using plink. SNPs with r^2^ > 0.6 were considered dependent and were called clusters.

## Results

### Characteristics of the study population

The median age at diagnosis was 67 (IQR 60–73) years, half of the population was male and the median BMI was 27 (IQR 24–30) kg/m^2^. Proximal colon tumors were most prevalent (38%), followed by distal colon (30%) and rectal cancers (22%) and unknown location (11%). The median dietary calcium intake in the total population was 694 g/day (IQR 467–995), 676 g/day (IQR 444; 993) for men and 706 (IQR 483; 997) for women. Almost 11% of the population used calcium supplements (>500 mg/day). Almost 40% of the population died, of which 4,914 (22%) were related to CRC, during follow-up time (median 4.8 years, IQR 2.4–8.4).

When comparing characteristics of participants with a low dietary calcium intake (quartile 1 and 2 of sex- and study-specific quartiles) with characteristics of participants with a high dietary calcium intake (quartile 3 and 4 of sex- and study-specific quartiles) the most striking differences observed were differences in the dietary intake. In general intake of energy, fiber, folate, fruit and vegetables was markedly lower in the low dietary calcium intake group compared to the high dietary calcium group. Detailed information can be found in Table 1. In addition, patients’ characteristics for each individual study can be found in Supplementary Table S2 and distribution of total and dietary calcium intake per study can be found in Supplementary Fig. S1.

**Table 1:** Characteristics of Colorectal cancer survivors for the total population and stratified by calcium intake.

	Total population	Low dietary calcium intake^a^	High dietary calcium intake^a^
	*N* = 18952	*N* = 6755	*N* = 6330
Age at diagnosis (years)	67.0 [60.0, 73.0]	69.0 [63.0, 75.0]	69.0 [62.0, 75.0]
Sex (male)	9516 (50)	3334 (49)	2905 (46)
BMI (kg/m^2^)	26.7 [24.1, 29.8]	26.6 [24.0, 29.4]	26.4 [24.0, 29.5]
Unknown	248	114	87
Self-reported race			
Caucasian	17885 (94)	6553 (97)	6186 (98)
Other	161 (1)	50 (1)	30 (0)
Unknown	906 (5)	152 (2)	114 (2)
Smoking status			
Current smoker	2351 (12)	859 (13)	693 (11)
Former smoker	7913 (42)	2917 (43)	2757 (44)
Never smoker	8064 (43)	2843 (42)	2764 (44)
Unknown	624 (3)	136 (2)	116 (2)
Education			
Very low	3458 (18)	1486 (22)	1076 (17)
Low	4289 (23)	1872 (28)	1548 (25)
Medium	4715 (25)	1494 (22)	1554 (25)
High	5608 (30)	1835 (27)	2080 (33)
Unknown	882 (5)	68 (1)	72 (1)
Dietary calcium intake (mg/day)	694 [467, 995]	488 [334, 653]	1001 [752, 1267]
Unknown	5867	0	0
Calcium supplement use			
<1 pill (<500 mg/day)	9755 (52)	2133 (32)	2168 (34)
≥1 pill (≥500 mg/day)	2082 (11)	758 (11)	911 (14)
Unknown	7115 (38)	3864 (57)	3251 (51)
Total calcium intake^c^ (mg/day)	991 [658, 1457]	658 [484, 990]	1294 [978, 1719]
Unknown	12982	3864	3251
Energy intake (kcal/day)	1843 [1430, 2347]	1590 [1 (232, 2003]	2123 [1701, 2702]
Unknown	9109	1822	1420
Total folate intake (µg/day)	485 [236, 895]	353 [245, 848]	554 [361, 1095]
Unknown	2878	1627	1251
Fiber intake (g/day)	20 [14, 26]	17 [13, 22]	22 [17, 30]
Unknown	9057	1790	1400
Red meat intake (portion/day)	0.6 [0.3, 0.9]	0.7 [0.4, 1.0]	0.7 [0.4, 1.1]
Unknown	415	93	73
Processed meat intake (portion/day)	0.2 [0.1, 0.6]	0.3 [0.1, 0.6]	0.3 [0.1, 0.6]
Unknown	3037	96	68
Vegetable intake (portion/day)	1.5 [1.0, 3.0]	1.7 [1.1, 3.0]	2.2 [1.1, 3.9]
Unknown	411	109	87
Fruit intake (portion/day)	1.4 [0.9, 2.5]	1.0 [0.6, 2.0]	1.8 [1.0, 2.8]
Unknown	477	122	98
Alcohol (g/day)	3.8 [0.0, 16.0]	3.9 [0.0, 16.5]	2.9 [0.0, 13.5]
Unknown	338	46	45
Family history			
No	11297 (60)	4410 (65)	4161 (66)
Yes	2624 (14)	844 (13)	855 (14)
Unknown	5031 (27)	1501 (22)	1314 (21)
Stage of disease			
Stage 1 or local	3654 (19)	1580 (23)	1586 (25)
Stage 2/3 or regional	7325 (39)	3170 (47)	2959 (47)
Stage 4 or distant	1625 (9)	748 (11)	653 (10)
Unknown	6348 (34)	1257 (19)	1132 (18)
Tumor location			
Distal colon	5649 (30)	2051 (30)	1881 (30)
Proximal colon	7116 (38)	2632 (39)	2700 (43)
Rectum	4192 (22)	1441 (21)	1177 (19)
Unknown	1995 (11)	631 (9)	572 (9)
Aspirine use			
No	6655 (35)	2650 (39)	2192 (35)
Yes	3551 (19)	1375 (20)	1253 (20)
Unknown	8746 (46)	2730 (40)	2885 (46)
NSAID use			
No	7957 (42)	3099 (46)	2620 (41)
Yes	1274 (7)	389 (6)	377 (6)
Unknown	9721 (51)	3267 (48)	3333 (53)
Diabetes			
No	15699 (83)	5442 (81)	5071 (80)
Yes	1707 (9)	581 (9)	563 (9)
Unknown	1546 (8)	732 (11)	696 (11)
CRC-specific deaths			
Yes	4194 (22)	1490 (22)	1398 (22)
Unknown	372 (2)	112 (2)	95 (2)
Deaths	6801 (36)	2404 (36)	2249 (36)
Cohort (Acronym)			
CCFR	3564 (19)	402 (6)	431 (7)
CPSII	1453 (8)	781 (12)	672 (11)
DACHS	2878 (15)	1627 (24)	1251 (20)
DALS	1115 (6)	568 (8)	547 (9)
EPIC	2025 (11)	1108 (16)	917 (15)
HPFS	358 (2)	197 (3)	161 (3)
MCCS	784 (4)	397 (6)	387 (6)
NHS	594 (3)	328 (5)	266 (4)
NSHDS	305 (2)	104 (2)	92 (2)
PHS	312 (2)	163 (2)	149 (2)
PLCO	913 (5)	449 (7)	464 (7)
UKB	2994 (16)	0 (0)	0 (0)
VITAL	280 (2)	134 (2)	116 (2)
WHI	1377 (7)	497 (7)	877 (14)

Values presented are median [quartile 1 – quartile 3] or number (percentage).

^a^Low dietary calcium intake was defined as quartile 1 and 2 of sex- and cohort- specific quartiles and a high dietary calcium intake was defined as quartile 3 and 4 of sex- and cohort-specific quartiles.

^b^Very low: less than high school graduate; low: high school graduate or completed GED; medium: some college or technical school; high: college graduate or graduate degree.

^c^total calcium intake is only calculated when data of both dietary as well as supplemental calcium intake was available.

### Associations between dietary, supplemental and total calcium intake in relation to CRC-specific and all-cause mortality

**Fig. 1:**
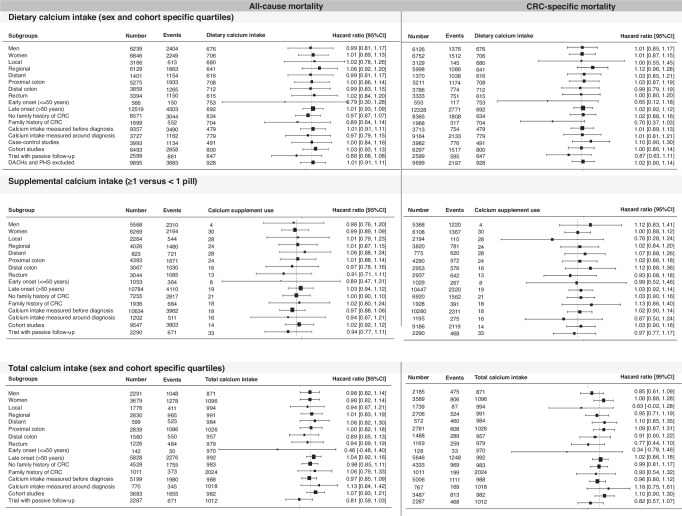
Associations between calcium intake and mortality in CRC survivors stratified by sex, stage of disease, tumor location, age at diagnosis, family history, timing of calcium intake and study design. The left sight of the figure shows the associations with all-cause mortality as the outcome, the right side of the figure for CRC-specific mortality. Analyses were done for dietary calcium (top 2 panels), supplemental calcium (middle 2 panels) and total calcium intake (bottom 2 panels).

For all-cause mortality, no associations were observed for the highest compared to the lowest sex- and study-specific quartile of dietary (HR 1.00, 95%CI 0.92–1.09), supplemental (HR 0.97, 95%CI 0.89–1.06) and total calcium intake (HR 0.99, 95%CI 0.88–1.11) (Table 2); similar patterns were noted for CRC-specific mortality. In addition, no associations were observed in the subgroup analyses, based on sex, tumor location, stage of disease, age at diagnosis, timing of calcium intake or study design (Fig. 1). Dietary calcium intake seems to be associated with CRC-specific mortality in persons with a family history of CRC (HR 0.70 95%CI 0.37–1.03). Timing of assessment of calcium intake did not influence the association between calcium intake and mortality (HR Q4 versus Q1 of dietary calcium intake 1.00 95%CI 0.91–1.10 for all-cause mortality and HR 1.01 95%CI 0.89–1.14 for CRC-specific mortality). For dietary and total calcium intake no heterogeneity between studies was observed (I^2^ 0–7%). For supplemental calcium intake in relation to all-cause mortality, moderate heterogeneity between included studies was observed (I^2^ 30%) (Supplementary data Figure S2).

**Table 2:** Associations between dietary calcium intake, supplemental calcium intake and total calcium intake in relation to CRC-specific and all-cause mortality in CRC survivors.

Dietary calcium intake					
All-cause mortality					
Sex- and cohort- specific quartiles	Quartile 1	Quartile 2	Quartile 3	Quartile 4	*P* for trend
Number/events	3368/1216	3387/1188	3157/1124	3173/1125	
HR (95%CI)	1.0 (ref)	0.97 (0.89–1.05)	0.99 (0.91–1.07)	1.00 (0.92–1.09)	0.862
CRC-specific mortality					
Number/events	3298/745	3345/745	3106/689	3129/709	0.824
HR (95%CI)	1.00 (Ref)	0.98 (0.88–1.08)	1.00 (0.90–1.10)	1.01 (0.91–1.12)	
Supplemental calcium intake					
All-cause mortality					
Supplement use	<1 pill	≥1 pill			
Number/events	9755/3708	2082/766			
HR (95%CI)	1.00 (Ref)	0.97 (0.89–1.06)			
CRC-specific mortality					
Supplement use	<1 pill	≥1 pill			
Number/events	9399/2111	2077/476			
HR (95%CI)	1.00 (Ref)	1.01 (0.90–1.13)			
Total calcium intake (dietary and supplemental intake)					
All-cause mortality					
Sex- and cohort- specific quartiles	Quartile 1	Quartile 2	Quartile 3	Quartile 4	*P* for trend
Number/events	1501/602	1487/573	1436/535	1546/616	
HR (95%CI)	1 (Ref)	0.98 (0.88–1.10)	0.95 (0.85–1.07)	0.99 (0.88–1.11)	0.735
CRC-specific mortality					
Number/events	1450/338	1440/313	1388/287	1496/434	
HR (95%CI)	1.0 (ref)	0.93 (0.80–1.08)	0.88 (0.75–1.03)	0.99 (0.85–1.15)	0.707

### Associations between genetic variants in the Calcium Sensing Receptor gene in relation to CRC-specific and all-cause mortality

Two related SNPs in the CaSR gene (rs62269066 and rs17282015) were statistically significantly associated with all-cause mortality after correction for multiple testing (Table S4). For both SNPs, a homozygous genotype for the alternative allele was associated with a lower risk of all-cause mortality (HR 0.86 95%CI 0.79–0.94). No statistically significant associations between SNPs and CRC-specific mortality were observed (Table S5). The 10 SNPs most significantly associated with CRC outcomes are depicted in Tables S4 and S5.

### Interaction between calcium intake and CaSR genetic variants in relation to CRC-specific and all-cause mortality

No statistically significant interactions were observed for dietary or total calcium intake and genetic variants in the *CaSR* gene in relation to all-cause or CRC-specific mortality (Table 3). On the contrary, multiplicative interaction between supplemental calcium intake and genetic variants in the *CaSR* gene was observed in relation to both all-cause mortality (independent SNP rs11713280 and correlated SNPs: rs62269066, rs11708053, rs11711698 and rs17282015) as well as CRC-specific mortality (rs11713280) (Table 3). Also, additive interaction between supplemental calcium intake and genetic variants in the *CaSR* gene was observed in relation to all-cause mortality (independent SNPs rs11713280 and rs62269066 and correlated SNPs: rs6780443, rs1782008 and rs7637874).

**Table 3:** Top 5 SNPS for which interaction between calcium intake and that specific genetic variant in relation to CRC outcomes was observed.

MULTIPLICATIVE INTERACTIONS											
Interaction GxE (calcium and CaSR)											
All-cause mortality						CRC-specific mortality					
Dietary calcium sex- and cohort- specific quartiles						Dietary calcium sex- and cohort- specific quartiles					
SNP	N/events	RERI^a^	95%CI	*P*-value^b^	MAF	SNP	N/events	RERI^a^	95%CI	*P*-value^b^	MAF
3:121910706_A/G rs1553309	11173/3841	1.04	1.00–1.08	0.075	0.42	3:121903842_C/A rs6780443^d^	11173/2499	1.08	1.02–1.14	0.012	0.24
3:121914667_T/C rs6764205^c^	11173/3841	1.04	1.00–1.09	0.049	0.30	3:121909830_C/G rs17282008^d^	11173/2499	1.07	1.01–1.14	0.018	0.24
3:121921700_T/C rs46780431219^c^	11173/3841	1.05	1.00–1.09	0.048	0.30	3:121932422_C/T rs62269066^e^	11173/2499	1.06	1.00–1.11	0.038	0.36
3:121922863_A/G rs4678045^c^	11173/3841	1.05	1.00–1.09	0.048	0.30	3:121936453_G/A rs11713280	11173/2499	1.12	1.01–1.23	0.026	0.07
3:121936453_G/A rs11713280	11173/3841	1.09	1.01–1.18	0.023	0.07	3:121966952_C/T rs7637874^d^	11173/2499	1.07	1.01–1.13	0.031	0.24
**Supplement ≥ 1 versus < 1 pill**						**Supplement ≥ 1 versus < 1 pill**					
3:121913708_C/T rs17282015^e^	9207/3210	1.24	1.09–1.42	0.0009	0.36	3:121913708_C/T rs17282015^e^	9207/1999	1.25	1.07–1.47	0.006	0.36
3:121917298_T/C rs11708053^e^	9207/3210	1.25	1.10–1.42	0.0007	0.36	3:121917298_T/C rs11708053^e^	9207/1999	1.26	1.07–1.47	0.005	0.36
3:121917387_A/G rs11711698^e^	9207/3210	1.25	1.10–1.42	0.0007	0.36	3:121917387_A/G rs11711698^e^	9207/1999	1.25	1.07–1.47	0.005	0.36
3:121932422_C/T rs62269066^e^	9207/3210	1.25	1.10–1.43	0.0006	0.36	3:121932422_C/T rs62269066^e^	9207/1999	1.24	1.06–1.46	0.008	0.36
3:121936453_G/A rs11713280	9207/3210	1.49	1.19–1.87	0.0006	0.07	3:121936453_G/A rs11713280	9207/1999	1.6	1.21–2.11	0.0009	0.07
**Total calcium intake sex- and cohort- specific quartiles**						**Total calcium intake sex- and cohort- specific quartiles**					
3:121992825_C/T rs3804594^f^	4646/1695	0.87	0.78–0.97	0.011	0.08	3:121994941_G/A rs2279802^f^	4646/1005	0.81	0.70–0.94	0.005	0.08
3:121995415_C/T rs13093602^f^	4646/1695	0.87	0.78–0.97	0.013	0.08	3:121995415_C/T rs13093602^f^	4646/1005	0.81	0.70–0.94	0.005	0.08
3:121996810_T/A rs1827381^f^	4646/1695	0.87	0.78–0.97	0.013	0.08	3:121996810_T/A rs1827381^f^	4646/1005	0.81	0.70–0.94	0.005	0.08
3:121997268_T/C rs3792291^f^	4646/1695	0.87	0.78–0.97	0.013	0.08	3:121997268_T/C rs3792291^f^	4646/1005	0.81	0.70–0.94	0.005	0.08
3:121999059_G/A rs2055427^f^	4646/1695	0.87	0.78–0.97	0.012	0.08	3:121999059_G/A rs2055427^f^	4646/1005	0.81	0.70–0.94	0.005	0.08

ADDITIVE INTERACTIONS											
Dietary calcium sex- and cohort- specific quartiles						Dietary calcium sex- and cohort- specific quartiles					
3:121921700_T/C rs4678043^c^	11173/3841	0.04	−0.00–0.08	0.068	0.30	3:121903842_C/A rs6780443^d^	11173/2499	0.06	0.02–0.11	0.013	0.24
3:121922863_A/G rs4678045^c^	11173/3841	0.04	−0.00–0.09	0.070	0.30	3:121909830_C/G rs17282008^d^	11173/2499	0.06	0.01–0.11	0.018	0.24
3:121936453_G/A rs11713280	11173/3841	0.07	0.02–0.13	0.015	0.07	3:121936453_G/A rs11713280	11173/2499	0.09	0.03–0.15	0.012	0.07
3:121996235_G/A rs77663377	11173/3841	0.07	−0.01–0.14	0.080	0.15	3:121966952_C/T rs7637874^d^	11173/2499	0.06	0.01–0.10	0.037	0.24
3:122005273_A/G rs34042920	11173/3841	0.04	−0.01–0.10	0.107	0.10	3:121996235_G/A rs77663377	11173/2499	0.09	0.003–0.17	0.051	0.15
**Supplement ≥ 1 versus < 1 pill**						**Supplement ≥ 1 versus < 1 pill**					
3:121903842_C/A rs6780443^d^	9207/3210	0.22	0.09–0.36	0.0009	0.24	3:121903842_C/A rs6780443^d^	9207/1999	0.23	0.05–0.41	0.010	0.24
3:121909830_C/G rs17282008^d^	9207/3210	0.22	0.09–0.36	0.0009	0.24	3:121909830_C/G rs17282008^d^	9207/1999	0.23	0.05–0.41	0.009	0.24
3:121932422_C/T rs62269066^e^	9207/3210	0.21	0.10–0.32	0.00006	0.36	3:121917298_T/C rs11708053^e^	9207/1999	0.22	0.07–0.36	0.003	0.36
3:121936453_G/A rs11713280	9207/3210	0.40	0.15–0.64	0.0009	0.07	3:121936453_G/A rs11713280	9207/1999	0.51	0.18–0.84	0.002	0.07
3:121966952_C/T rs7637874^d^	9207/3210	0.22	0.08–0.36	0.0009	0.24	3:121966952_C/T rs76377874^d^	9207/1999	0.22	0.04–0.09	0.015	0.24
**Total calcium intake sex- and cohort- specific quartiles**						**Total calcium intake sex- and cohort- specific quartiles**					
3:121984792_G/A rs17197671^g^	4646/1695	0.07	0.004–0.13	0.015	0.14	3:121930454_C/G rs75473459^h^	4646/1005	0.10	0.02–0.18	0.021	0.11
3:121988851_G/C rs6783855^g^	4646/1695	0.07	0.004–0.13	0.015	0.14	3:121917298_T/C rs11708053^e^	4646/1005	0.08	0.01–0.15	0.030	0.36
3:121990800_T/C rs1604446^g^	4646/1695	0.07	0.004–0.13	0.015	0.14	3:121917387_A/G rs11711698^e^	4646/1005	0.08	0.01–0.15	0.032	0.36
3:122002178_T/C rs2134224^g^	4646/1695	0.07	0.003–0.13	0.014	0.14	3:121939743_C/T rs36004543^h^	4646/1005	0.11	0.03–0.19	0.004	0.12
3:122005273_A/G rs34042920^g^	4646/1695	0.09	0.03–0.16	0.005	0.10	3:121958475_G/A rs62269089^h^	4646/1005	0.10	0.01–0.18	0.020	0.11

^a^Models were adjusted for age at diagnosis, sex, cohort, and the first three principal components of genetic ancestry as well as main effects of calcium intake (dietary, supplemental or total) and SNPs. Sex- and cohort- specific quartiles as well as SNPs were entered as continuous variables for both the multiplicative as the additive interaction models.

^b^The estimated effective number of independent tests among 325 SNPS was 35 based on the simple M approach. Therefore, *p* < 0.001 were considered statistically significant (*n* = 35; 0.05/35 = 0.001).

^c,d,e,f,g,h^Correlated SNPS (r^2^ > 0.6).

We further explored the two independent SNPs (rs11713280 and rs62269066) for which both additive and multiplicative interaction with supplemental calcium intake were observed in relation to all-cause mortality (*p* multiplicative interaction = 0.0006 and 0.0006; *p* additive interaction = 0.0009 and 0.0006 and CRC-specific mortality (*p* multiplicative interaction = 0.0009 and 0.008; *p* additive interaction = 0.002 and 0.004, respectively (Table 4). For rs11713280, the association between supplemental calcium intake and all-cause mortality differed between persons who were homozygous for the reference allele (GG) (HR 0.93 95%CI 0.84–1.04) compared to persons who were heterozygous (GA) (HR 1.35 95%CI 1.07–1.70) or for the homozygous alternative allele (AA) (HR 3.83 95%CI 1.23–11.96). A similar trend was observed for CRC-specific mortality (GG: HR 0.97 95%CI 0.85–1.11; GA 1.50 95%CI 1.13–1.99; AA: HR 3.92 95%CI 1.05–14.72). Also, different associations for supplemental calcium intake in relation to mortality were observed depending on polymorphism of rs62269066. Persons who were homozygous for the reference allele (CC) had a lower risk of all-cause mortality when taking supplements (HR 0.80 95%CI 0.69-0.93), while persons with a CT or TT genotype had a non-significant higher risk of all-cause mortality (HR 1.12 95%CI 0.98–1.28 and HR 1.19 95%CI 0.92–1.53, respectively). A similar trend was observed for CRC-specific mortality (CC: HR 0.85 95%CI 0.71–1.03; CT 1.20 95%CI 1.02–1.43; TT: HR 1.23 95%CI 0.90–1.67).

**Table 4:** Elaborate example Multiplicative and Additive interactions of two SNPs in the CaSR gene and CRC outcomes

ALL-CAUSE MORTALITY				COLORECTAL CANCER SPECIFIC MORTALITY			
SUPPLEMENTAL CALCIUM INTAKE*3:121936453_G/A (rs11713280)				SUPPLEMENTAL CALCIUM INTAKE**3:121936453_G/A (rs11713280)			
	<1 pill	≥1 pill	Effect of supplement intake within the strata of ‘3:121936453_G/A‘		<1 pill	≥1 pill	Effect of supplement intake within the strata of ‘3:121936453_G/A‘
3:121936453_G/A	Number/events HR [95% CI]	Number/events HR [95% CI]	Number/events HR [95% CI]	3:121936453_G/A	Number/events HR [95% CI]	Number/events HR [95% CI]	Number/events HR [95% CI]
GG	6493/2276 Ref	1436/496 0.93 (0.84–1.04)	7929/2772 0.93 (0.84–1.04)	GG	6493/1410 Ref	1436/316 0.97 (0.85–1.11)	7929/1726 0.97 (0.85–1.11)
GA	1003/326 0.86 (0.76–0.96)	237/98 1.15 (0.93–1.41)	1240/424 1.35 (1.07–1.70)	GA	1003/196 0.88 (0.76–1.02)	237/68 1.31 (1.02–1.69)	1240/264 1.50 (1.13–1.99)
AA	31/9 0.64 (0.33–1.24)	7/5 2.13 (0.88–5.13)	38/14 3.83 (1.23–11.96)	AA	31/5 0.67 (0.28–1.62)	7/4 2.57 (0.96–6.89)	38/9 3.92 (1.05–14.72)
Effect of ‘3:121936453_G/A‘ within the strata of supplement intake	7527/2611 0.85 (0.76–0.95)	1680/599 1.27 (1.04–1.55)		Effect of ‘3:121936453_G/A‘ within the strata of supplement intake	7527/1611 0.87 (0.76–1.00)	1680/388 1.39 (1.09–1.77)	
Multiplicative scale	1.49 (1.19–1.87) *P* = 0.0006			Multiplicative scale	1.60 (1.21–2.11) *P* = 0.0009		
RERI	0.40 (0.15–0.64) *P* = 0.0009			RERI	0.51 (0.18–0.84) *P* = 0.002		

SUPPLEMENTAL CALCIUM INTAKE*3:121932422_C/T (rs62269066)				SUPPLEMENTAL CALCIUM INTAKE*3:121932422_C/T (rs62269066)			
	<1 pill	≥1 pill	Effect of supplement intake within the strata of ‘3:121932422_C/T‘		<1 pill	≥1 pill	Effect of supplement intake within the strata of ‘3:121932422_C/T ‘
3:121932422_C/T	Number/events HR [95% CI]	Number/events HR [95% CI]	Number/events HR [95% CI]	3:121932422_C/T	Number/events HR [95% CI]	Number/events HR [95% CI]	Number/events HR [95% CI]
CC	3113/1130 Ref	706/223 0.81 (0.69–0.94)	3819/1353 0.80 (0.69–0.93)	CC	3113/707 Ref	706/145 0.85 (0.71–1.03)	3819/852 0.85 (0.71–1.03)
CT	3476/1193 0.90 (0.83–0.98)	747/296 1.01 (0.88–1.16)	4223/1486 1.12 (0.98–1.28)	CT	3476/724 0.90 (0.81–0.99)	747/190 1.08 (0.91–1.28)	4223/911 1.20 (1.02–1.43)
TT	938/288 0.78 (0.68–0.89)	227/80 0.93 (0.74–1.17)	1165/368 1.19 (0.92–1.53)	TT	938/180 0.81 (0.69–0.96)	227/53 0.99 (0.75–1.32)	1165/233 1.23 (0.90–1.67)
Effect of ‘3:121932422_C/T‘ within the strata of supplement intake	7527/2611 0.89 (0.84–0.94)	1680/599 1.11 (0.99–1.25)		Effect of ‘3:121932422_C/T ‘ within the strata of supplement intake	7527/1611 0.90 (0.84–0.97)	1680/388 1.11 (0.97–1.29)	
Multiplicative scale	1.25 (1.10–1.43) *P* = 0.0006			Multiplicative scale	1.24 (1.06–1.46) *P* = 0.008		
RERI	0.21 (0.10–0.32) *P* = 0.0006			RERI	0.21 (0.06–0.35) *p* = 0.004		

Models were adjusted for age at diagnosis, sex, cohort, and the first three principal components of genetic ancestry and for the interaction models also for the main effects of calcium intake (dietary, supplemental or total) and SNP.

The estimated effective number of independent tests among 325 SNPS was 35 based on the simple M approach. Therefore, *p* < 0.001 were considered statistically significant (*n* = 35; 0.05/35 = 0.001).

A RERI of zero should be interpret as no additive interaction, a RERI < 0 a negative additive interaction and a RERI > 0 a positive additive interaction.

Total calcium intake is not included in this table because of the small sample sizes in the subgroups (*n* = 4646 for total population with total calcium intake available).

*Median split sex- and cohort-specific dietary calcium intake.

## Discussion

In this large consortium of CRC patients, no association between dietary, supplemental or total calcium intake in relation to all- cause mortality nor CRC-specific mortality was observed. Two SNPs in the *CaSR* gene were associated with all-cause mortality after correction for multiple testing, while no associations between SNPs in the *CaSR* gene and CRC-specific mortality were observed. In addition, no interactions between dietary calcium intake or total calcium intake and SNPs in the *CaSR* gene in relation to mortality were observed. However, multiplicative interactions were observed between supplemental calcium intake and 5 SNPs, of which 2 independent clusters, in relation to all-cause mortality and 1 SNP in relation to CRC-specific mortality. In addition, additive interactions were observed between supplemental calcium intake and 5 SNPs, of which 3 independent clusters, in relation to all-cause mortality.

In our study we did not observe an association between dietary, supplemental or total calcium intake and all-cause or CRC-specific mortality in CRC patients. This is against our initial hypothesis, since we expected a better survival rate with a higher calcium intake given the associations of calcium intake with CRC risk and mortality [1, 45]. However, our results are consistent with previous studies investigating pre-diagnostic calcium intake and CRC survival, which also did not observe associations in CRC patients (*n* = 148–3859), with HRs ranging from 0.63–1.35 [6–9, 11]. Three of the previously mentioned analyses [6–8] included study populations that also participated in the ISACC consortium (NHS/HPFS; EPIC; CPSII), and part of their data is used in these analyses. We now hypothesize that the timing of calcium intake is of importance. For the data available for the present analyses, calcium intake was assessed before or around diagnosis (median 2.0 years before diagnosis IQR 1–6 years before diagnosis); however, it is possible that post-diagnostic intake may be most relevant to outcomes. Previous studies have noted an inverse association between post-diagnostic calcium intake and CRC-specific and all-cause mortality [6, 8, 10]. This was only statistically significant in NHS/HPFS for CRC-specific mortality (total calcium intake HR _quartile 4 vs quartile 1_ 0.56 95%CI 0.32–0.96) [6] and in CPSII for all-cause mortality (total calcium intake HR _quartile 4 vs quartile 1_ 0.72 95%CI 0.53–0.98) [8]. In addition, previous studies investigating the effect of dietary calcium intake in normal colonic mucosa, found a direct upregulation of several genes, including FOXJ2 and C3aR1, indicating a rather short term effect of calcium intake [46]. As no associations were observed in the subgroup analyses either, calcium intake before or around diagnosis does not appear to be associated with survival after a CRC diagnosis. Thus, although calcium intake before diagnosis does not improve CRC outcomes, it also does not hamper prognosis and thus can be consumed safely. Further studies should focus on post-diagnostic calcium intake and/or more long-term calcium exposure, for example by measuring calcium intake multiple times during follow-up. Also, sources of calcium intake, e.g., dairy or vegetables, as well as nutrients known to interact with calcium homeostasis such as vitamin D and magnesium [47] should be considered in these further studies. Different sources of calcium may have different effects on the gut microbiome [48] and likely subsequently the tumor microenvironment and immune-response [49] and could thus differentially influence survival.

Two correlated SNPs (rs62269066 and rs17282015) were associated with all-cause mortality, where a genotype of two minor alleles was associated with a lower risk of all-cause mortality for both SNPs. The function of these two SNPs is unknown and both SNPs are intron variants. No SNPs in the *CaSR* gene were associated with CRC-specific mortality after correction for multiple testing. To our knowledge, only four relatively small studies (ranging from 531 to 1,202 participants) have previously investigated the association between *CaSR* polymorphism and CRC outcomes [16, 50–52]. The *CaSR* rs1801725 SNP was associated with overall survival in a case-control study in China [52], but was not associated with survival in the an European cohort [51]. In addition, this SNP seems to be associated with CRC recurrence in a Hungarian population [50]. This specific SNP was not associated with mortality in the current study. Thus, although there are some indications that certain SNPs in the *CaSR* gene are associated with CRC prognosis, these SNPS are not confirmed in multiple studies.

In line with our results, a study in a Canadian population (*n* = 531 CRC patients) did not observe an interaction between dietary calcium intake and *CaSR* polymorphisms in relation to overall or disease-free survival after correction for multiple testing [16]. While no interaction was shown for dietary intake of calcium, we did observe an interaction with supplemental intake of calcium. To our knowledge, no previous studies investigated multiplicative and additive interactions between supplemental calcium intake and genetic variants of the *CaSR* in relation to CRC outcomes. Based on our results, there seems to be an interaction between supplemental calcium intake (>500 mg/day) and genetic variants in the *CaSR* gene in relation to all-cause mortality and CRC-specific mortality. Although we expected these interactive effects of CaSR also for dietary calcium intake, it could be that higher calcium levels, as with supplemental intake, are needed to exert these effects. In our population the median dietary calcium intake was 694 [IQR 467–995] mg/day, while the recommended daily intake is 1000–1200 mg/day (depending on age and sex) [53]. With supplemental intake of calcium (1 pill was assumed to be 500 mg, although there is some variation) on top of dietary intake, this amount is easily reached. In our analyses, an interaction between 7 SNPs in the *CaSR* gene and supplemental calcium intake in relation to mortality was observed. Whether these 7 SNPs, or correlated SNPs, change function and activity of the CaSR is unknown, all SNPs were intron variants. To conclude, our findings need to be confirmed in other studies and underlying mechanisms as well as functions of these SNPs should be further investigated before we can either discourage or encourage calcium supplement use in CRC patients based on their genotype.

Besides the *CaSR* gene, which encodes for the CaSR that plays a critical role in calcium homeostasis and has several tumor suppressing functions [12, 15], many more genes could potentially influence regulation and functioning of enzymes involved in calcium homeostasis and metabolism as well as influence effects of calcium on cancer progression [54]. In this study, we used a candidate-gene approach to investigate whether calcium intake in relation to all-cause and CRC-specific mortality was modified by genetic variants of the *CaSR* gene, which could potentially affect the functioning of the CaSR. As a complementary method, further studies should consider a genome wide approach when investigating interactions between calcium intake and genetic variants on mortality. Besides, in addition to investigating more variants across the genome, it would be interesting to evaluate the intake of the nutrients closely related to or interacting with calcium homeostasis, such as magnesium and vitamin D, at the same time.

This study has several important strengths. First of all, this is the largest consortium of CRC patients to date, consisting of almost 19,000 persons. Second, both dietary and supplemental sources of calcium intake were investigated. Third, we had detailed information about demographic and clinical characteristics, thus we were able to investigate several subgroups and adjust for potential confounders. However, this study is not without limitations. Given the nature of this large consortium, dietary and supplemental intake of calcium as well as genetic variants were assessed using different methods. This could hamper the ability to detect true associations for example due to misclassification of calcium intake. To prevent this as much as possible we used sex- and study specific quartiles of calcium intake. Furthermore, we imputed all SNPs of all studies using the same reference panel and imputation server. In addition, we had no information available about dietary sources of calcium intake, e.g., dairy, nor about nutrients closely related to calcium homeostasis, such as magnesium and vitamin D. Although we observed that total calcium intake is not associated with survival, we do not know whether specific dietary sources of calcium intake or the relative contribution of calcium compared to other nutrients in the diet, for example, the calcium to magnesium ratio, influence CRC survival [55, 56]. Also, data on treatment received e.g., chemotherapy, radiotherapy, was lacking. Adding stage of disease and tumor location, which together are closely linked to treatment provided, did not change the results. Finally, our study only included individuals of European ancestry, limiting the generalizability of our findings to other racial/ethnic groups.

To conclude, calcium intake before and around diagnosis was not associated with all-cause or CRC-specific mortality. However, multiplicative as well as additive interactions between supplemental calcium intake and genetic variants in the *CaSR* in relation to all-cause mortality and CRC-specific mortality were observed. Further studies should focus on post-diagnostic calcium intake, and include sources of calcium as well as closely related nutrients, and should investigate interactions between calcium intake and genetic variants in relation to mortality using a genome-wide approach.

## Supplementary information

Supplementary Fig.s and Tables

Supplementary_tableS2

## Data Availability

The datasets generated during and/or analysed during the current study are available from the corresponding author on reasonable request.
