# Supplementary material for: Calcium intake and genetic variants in the calcium sensing receptor in relation to colorectal cancer mortality: an international consortium study of 18,952 patients
Source: BJC Rep. 2024 Sep 2;2:63. doi: 10.1038/s44276-024-00077-3 (PMC11368808; doi:10.1038/s44276-024-00077-3)
Supplement: Supplementary file 1 — Supplementary Fig.s and Tables [file 44276_2024_77_MOESM1_ESM.docx]

Supplementary tables belonging to the manuscript entitled: “Calcium intake and genetic variants in the calcium sensing receptor in relation to colorectal cancer mortality: an international consortium study of 18 952 patients”

The following tables can be found in this document.

Table S1: Characteristics included studies

Figure S1: Total and dietary calcium intake stratified per study

Table S2: Characteristics CRC survivors stratified per study (separate excel file)

Table S3: Characteristics included SNPs

Figure S2: Meta-analyses Calcium intake and CRC outcomes

Table S4 and S5: Top 10 SNPs CaSR associated with CRC outcomes

**Supplementary tables**

***Table S1:*** *Characteristics of included studies*

| Study acronym | Study design | Country of origin | Total number of participants; number of deaths / number of CRC-specific deaths * | Median follow-up time (days) | Male (%) | Age at diagnosis (years) | Calcium data available | Method dietary calcium is assessed | Genetic data platform | Assessment of clinical outcomes |
| --- | --- | --- | --- | --- | --- | --- | --- | --- | --- | --- |
| CCFR (31) | International consortium (cohort) in persons with high genetic risk of CRC | USA/ Canada/ Australasia | 3654;  1671/893 | 4037 | 54 | 57.8 (10.7) | Both dietary and supplemental intake | 119 items FFQ | Illumina 1M/1M-Duo, Omni1-Quad, Infinium OncoArray; Affymetrix Axiom | Linkage to state or national death registries, or state cancer registries, or review of medical records, or active follow-up of family members; dates and causes of death verified by death certificates. |
| CPSII (23,25) | Population based cohort | USA | 1453;  1133/320 | 2114 | 51 | 75.0 (6.4) | Both dietary and supplemental intake | FFQ modified from 60-items Health Habits and History Questionnaire | Axiom | Linkage to state death registries, or state cancer registries, with cause of death verified by death certificates. |
| DACHS (26,33) | Population based case-control study | Germany | 2878;  780/564 | 1182 | 59 | 68.3 (10.7) | Dietary intake | 23 item FFQ | OmniExpress, CytoSNP | Linkage to national death registries, or cancer registries, with cause of death verified by death certificates. |
| DALS  (24) | Population based case control study | USA | 1115;  354/212 | 1796 | 55 | 65.0 (9.9) | Dietary intake | Diet-history questionnaire | CytoSNP | Linkage to state death registries, or state cancer registries, with cause of death verified by death certificates. |
| EPIC  (28) | Population based cohort study | Europe | 2025;  647/505 | 1267 | 44 | 63.3 (8.1) | Dietary intake | Country-specific FFQs | OmniExpress + ExomeChip | Linkages with cancer registries, boards of health and death indices or active follow-up (mail or telephone to participants, municipal registries, health departments, physicians and hospitals) |
| HPFS  (30) | Cohort study in male health professionals | USA | 358;  210/86 | 3217 | 100 | 71.7 (8.8) | Both dietary and supplemental intake | +/- 160 item FFQ | OmniExpress + ExomeChip | Review of death certificates and/or medical records |
| MCCS  (29,34) | Population based cohort study | Australia | 784;  356/199 | 4226 | 52 | 70.7 (8.9) | Dietary intake | 80 item FFQ (The DQES v3.2) | Axiom, OncoArray | Linkage to national death registries, or cancer registries, with cause of death verified by death certificates. |
| NHS  (27) | Cohort study in female nurses | USA | 594;  247/156 | 2839 | 0 | 69.1 (8.6) | Both dietary and supplemental intake | 105 item FFQ | OmniExpress, OmniExpress + ExomeChip | Active follow-up with dates and causes of death confirmed via review of death certificates and/or medical records |
| NSHDS  (35,38) | Population cohort | Sweden | 305;  158/- | 2624 | 51 | 62.9 (8.2) | Both dietary and supplemental intake | 64 or 84 items FFQ depending on the year of assessment. Validated: (17) |  | Linkage to national death registries, or cancer registries |
| PHS  (19) | Trial (aspirin in CVD) with passive follow-up cohort afterwards  Males | USA | 312;  190/127 | 2045 | 100 | 70.9 (9.6) | Dietary intake | 19 item FFQ | OmniExpress | Active follow-up with dates and causes of death confirmed via review of death certificates and/or medical records |
| PLCO (36) | Trial (screening) | USA | 913;  251/161 | 1917 | 57 | 69.7 (6.3) | Both dietary and supplemental intake | 124 item Diet History Questionnaire | InitialGwas, CytoSNP | Linkage to state death registries, or state cancer registries, with cause of death verified by death certificates. |
| UKB  (18,37) | Population cohort (UK biobank) | United Kingdom | 2994;  793/594 | 1119 | 58 | 64.4 (6.5) | Supplemental intake | Dietary questionnaire with 29 questions on diet. | Axiom | Linkage to national death registries or cancer registries. |
| VITAL  (22) | Population cohort (supplement use and cancer risk). | USA | 280;  115/70 | 1839 | 53 | 69.8 (6.6) | Both dietary and supplemental intake | 120 item FFQ with additional questions about fortified foods. | CytoSNP | Linkage with state death registries and active by family members. |
| WHI  (17,20,21) | Clinical trial and cohort in post-menopausal women. | USA | 1377;  420/307 | 1392 | 0 | 71.6 (7.2) | Both dietary and supplemental intake | 122 item FFQ | CytoSNP, InitialGwas | Reviewing death certificates, medical records, or autopsy reports, or by linkage to the state death registries |

*For total cohort, can be different for each specific analysis (i.e., dietary intake vs supplemental intake). FFQ= Food Frequency Questionnaire


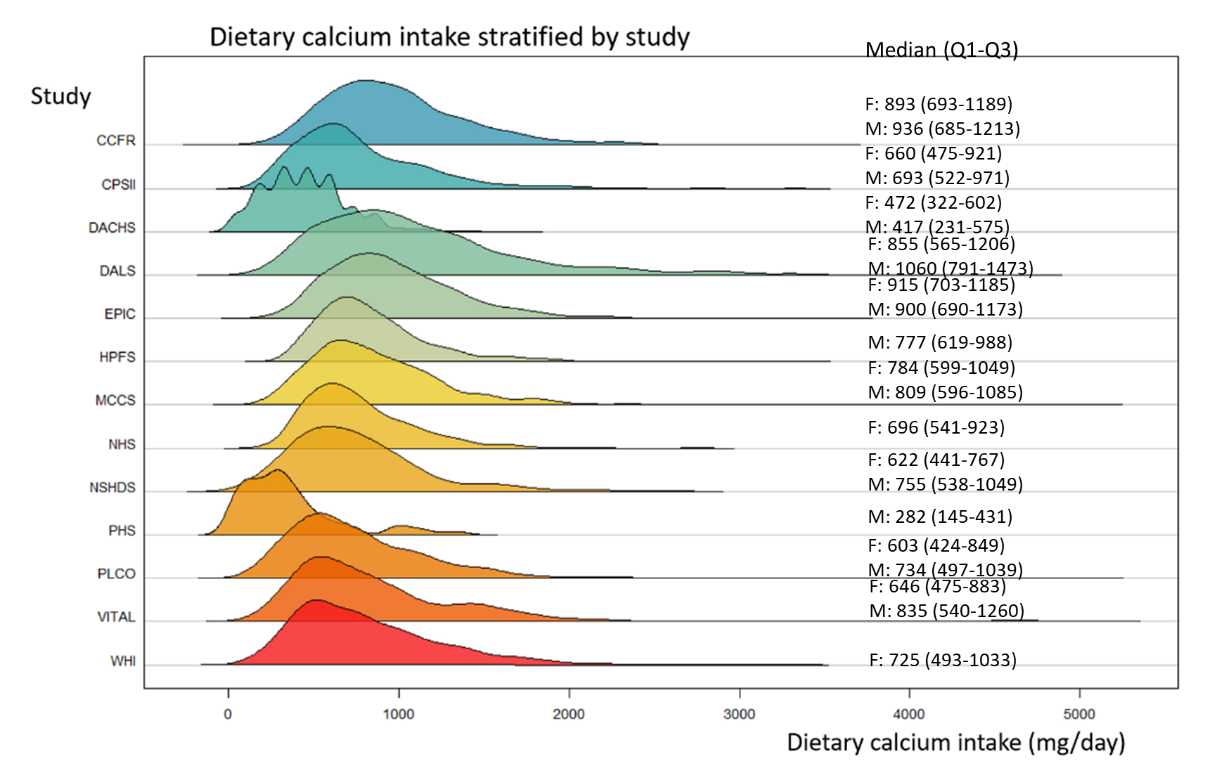
***Figure S1***

*
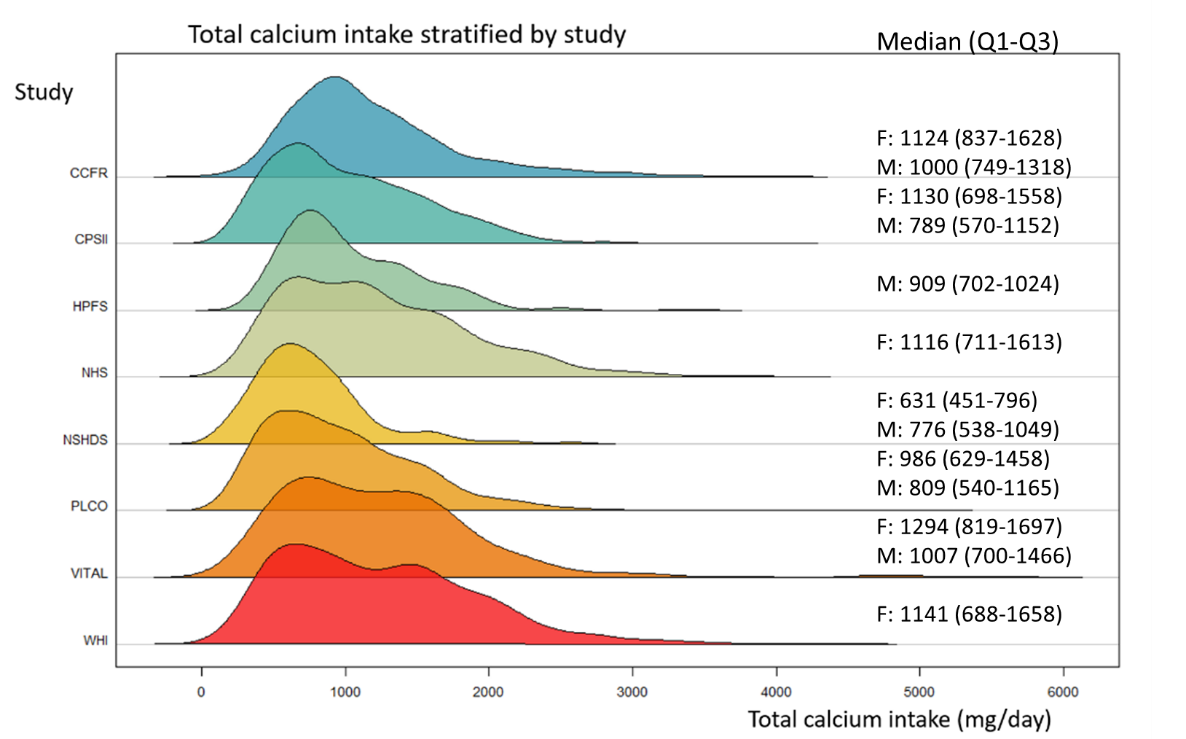
*

*Dietary and total calcium intake stratified by study. F=Female M=Male.*

***Table S3:*** *Characteristics of included SNPS*

| Gene | SNP | MAF | HWE (p-value) | Mean imputation quality (R2) |
| --- | --- | --- | --- | --- |
| CaSR | 3:121902907_T/C | 0.22 | 0.40 | 1.00 |
| CaSR | 3:121902945_C/A | 0.36 | 0.38 | 1.01 |
| CaSR | 3:121903195_C/G | 0.15 | 0.95 | 0.99 |
| CaSR | 3:121903266_G/A | 0.22 | 0.52 | 1.00 |
| CaSR | 3:121903836_T/A | 0.17 | 0.74 | 0.99 |
| CaSR | 3:121903842_C/A | 0.24 | 0.72 | 0.99 |
| CaSR | 3:121903936_A/T | 0.17 | 0.74 | 0.99 |
| CaSR | 3:121904211_G/T | 0.15 | 0.95 | 0.99 |
| CaSR | 3:121905363_C/A | 0.22 | 0.49 | 1.00 |
| CaSR | 3:121905657_A/G | 0.36 | 0.38 | 1.01 |
| CaSR | 3:121905761_G/C | 0.13 | 0.74 | 1.00 |
| CaSR | 3:121906501_G/A | 0.17 | 0.72 | 0.99 |
| CaSR | 3:121906653_G/A | 0.17 | 0.72 | 0.99 |
| CaSR | 3:121906812_T/C | 0.36 | 0.38 | 1.01 |
| CaSR | 3:121907328_A/G | 0.36 | 0.38 | 1.01 |
| CaSR | 3:121907476_A/G | 0.22 | 0.39 | 1.00 |
| CaSR | 3:121907494_G/A | 0.36 | 0.38 | 1.01 |
| CaSR | 3:121908434_T/C | 0.22 | 0.39 | 1.00 |
| CaSR | 3:121909291_C/T | 0.17 | 0.72 | 0.99 |
| CaSR | 3:121909776_G/T | 0.13 | 0.76 | 1.00 |
| CaSR | 3:121909830_C/G | 0.24 | 0.71 | 0.99 |
| CaSR | 3:121909888_A/G | 0.13 | 0.76 | 1.00 |
| CaSR | 3:121910005_G/T | 0.13 | 0.76 | 1.00 |
| CaSR | 3:121910127_A/G | 0.17 | 0.72 | 0.99 |
| CaSR | 3:121910628_T/A | 0.19 | 0.51 | 1.01 |
| CaSR | 3:121910672_A/G | 0.19 | 0.55 | 1.00 |
| CaSR | 3:121910695_C/G | 0.19 | 0.51 | 1.01 |
| CaSR | 3:121910706_A/G | 0.42 | 0.76 | 1.01 |
| CaSR | 3:121910937_A/T | 0.42 | 0.76 | 1.01 |
| CaSR | 3:121911070_A/C | 0.17 | 0.74 | 0.99 |
| CaSR | 3:121911520_A/G | 0.17 | 0.74 | 0.99 |
| CaSR | 3:121912016_C/T | 0.17 | 0.74 | 0.99 |
| CaSR | 3:121913370_G/T | 0.22 | 0.39 | 1.00 |
| CaSR | 3:121913374_G/C | 0.17 | 0.74 | 0.99 |
| CaSR | 3:121913708_C/T | 0.36 | 0.71 | 1.00 |
| CaSR | 3:121914396_A/T | 0.13 | 0.76 | 0.99 |
| CaSR | 3:121914414_C/A | 0.13 | 0.79 | 1.00 |
| CaSR | 3:121914667_T/C | 0.30 | 0.44 | 1.01 |
| CaSR | 3:121914706_T/C | 0.22 | 0.41 | 1.00 |
| CaSR | 3:121914857_G/A | 0.17 | 0.79 | 0.99 |
| CaSR | 3:121914952_G/A | 0.17 | 0.79 | 0.99 |
| CaSR | 3:121915255_G/A | 0.47 | 0.98 | 1.01 |
| CaSR | 3:121915552_T/A | 0.08 | 0.37 | 1.02 |
| CaSR | 3:121916109_G/A | 0.17 | 0.85 | 1.00 |
| CaSR | 3:121916325_G/A | 0.08 | 0.37 | 1.02 |
| CaSR | 3:121916544_T/C | 0.08 | 0.37 | 1.02 |
| CaSR | 3:121916686_C/A | 0.22 | 0.40 | 1.00 |
| CaSR | 3:121917298_T/C | 0.36 | 0.71 | 0.99 |
| CaSR | 3:121917319_A/C | 0.13 | 0.81 | 1.00 |
| CaSR | 3:121917387_A/G | 0.36 | 0.70 | 0.99 |
| CaSR | 3:121918283_C/T | 0.17 | 0.81 | 0.99 |
| CaSR | 3:121918416_T/A | 0.08 | 0.37 | 1.02 |
| CaSR | 3:121918491_G/A | 0.47 | 0.95 | 1.01 |
| CaSR | 3:121919740_G/A | 0.22 | 0.41 | 1.00 |
| CaSR | 3:121919987_C/A | 0.17 | 0.85 | 0.99 |
| CaSR | 3:121920426_G/A | 0.17 | 0.85 | 0.99 |
| CaSR | 3:121920569_G/A | 0.17 | 0.81 | 0.99 |
| CaSR | 3:121921084_C/T | 0.13 | 0.79 | 1.00 |
| CaSR | 3:121921137_C/T | 0.13 | 0.79 | 1.00 |
| CaSR | 3:121921530_T/A | 0.17 | 0.79 | 0.99 |
| CaSR | 3:121921700_T/C | 0.30 | 0.45 | 1.01 |
| CaSR | 3:121922222_C/G | 0.22 | 0.48 | 1.00 |
| CaSR | 3:121922253_C/A | 0.17 | 0.81 | 0.99 |
| CaSR | 3:121922795_C/T | 0.17 | 0.68 | 0.99 |
| CaSR | 3:121922863_A/G | 0.30 | 0.47 | 1.00 |
| CaSR | 3:121922879_T/A | 0.08 | 0.44 | 1.02 |
| CaSR | 3:121923033_T/G | 0.13 | 0.81 | 0.99 |
| CaSR | 3:121923326_G/A | 0.49 | 0.79 | 1.00 |
| CaSR | 3:121923878_G/C | 0.27 | 0.26 | 0.99 |
| CaSR | 3:121923997_G/T | 0.49 | 0.77 | 1.00 |
| CaSR | 3:121924384_G/T | 0.24 | 0.22 | 1.01 |
| CaSR | 3:121924625_T/G | 0.17 | 0.91 | 0.99 |
| CaSR | 3:121924998_A/G | 0.49 | 0.79 | 1.00 |
| CaSR | 3:121925198_G/C | 0.17 | 0.91 | 0.99 |
| CaSR | 3:121925212_G/C | 0.17 | 0.91 | 0.99 |
| CaSR | 3:121925572_G/A | 0.49 | 0.79 | 1.00 |
| CaSR | 3:121925798_G/T | 0.49 | 0.78 | 1.00 |
| CaSR | 3:121926820_C/T | 0.17 | 0.87 | 0.99 |
| CaSR | 3:121927745_G/A | 0.49 | 0.99 | 0.99 |
| CaSR | 3:121927927_G/A | 0.15 | 0.88 | 0.99 |
| CaSR | 3:121927941_C/G | 0.27 | 0.40 | 0.99 |
| CaSR | 3:121928025_G/A | 0.08 | 0.34 | 1.01 |
| CaSR | 3:121928476_A/G | 0.49 | 0.99 | 0.99 |
| CaSR | 3:121928682_T/C | 0.24 | 0.19 | 1.01 |
| CaSR | 3:121929073_A/C | 0.17 | 1.00 | 0.99 |
| CaSR | 3:121929112_A/G | 0.17 | 1.00 | 0.99 |
| CaSR | 3:121929207_G/T | 0.24 | 0.19 | 1.01 |
| CaSR | 3:121929287_G/A | 0.24 | 0.19 | 1.01 |
| CaSR | 3:121929441_A/C | 0.08 | 0.34 | 1.01 |
| CaSR | 3:121929450_A/G | 0.24 | 0.19 | 1.01 |
| CaSR | 3:121929686_G/A | 0.24 | 0.19 | 1.01 |
| CaSR | 3:121929725_A/G | 0.17 | 1.00 | 0.99 |
| CaSR | 3:121930306_A/G | 0.24 | 0.20 | 1.01 |
| CaSR | 3:121930454_C/G | 0.11 | 0.76 | 0.97 |
| CaSR | 3:121930499_A/G | 0.08 | 0.32 | 1.01 |
| CaSR | 3:121930520_C/T | 0.24 | 0.20 | 1.01 |
| CaSR | 3:121930729_A/G | 0.27 | 0.39 | 0.99 |
| CaSR | 3:121930771_G/A | 0.24 | 0.20 | 1.01 |
| CaSR | 3:121930788_A/G | 0.24 | 0.20 | 1.01 |
| CaSR | 3:121930840_C/A | 0.24 | 0.20 | 1.01 |
| CaSR | 3:121930974_C/T | 0.08 | 0.32 | 1.01 |
| CaSR | 3:121930999_C/T | 0.24 | 0.20 | 1.01 |
| CaSR | 3:121931228_A/G | 0.24 | 0.19 | 1.01 |
| CaSR | 3:121931348_G/A | 0.17 | 1.00 | 0.99 |
| CaSR | 3:121931395_A/C | 0.24 | 0.19 | 1.01 |
| CaSR | 3:121931571_C/T | 0.27 | 0.39 | 0.99 |
| CaSR | 3:121931709_A/G | 0.24 | 0.19 | 1.01 |
| CaSR | 3:121932092_G/A | 0.24 | 0.19 | 1.01 |
| CaSR | 3:121932197_G/C | 0.24 | 0.20 | 1.01 |
| CaSR | 3:121932422_C/T | 0.36 | 0.69 | 0.98 |
| CaSR | 3:121932457_G/A | 0.17 | 1.00 | 0.99 |
| CaSR | 3:121932534_A/G | 0.24 | 0.20 | 1.01 |
| CaSR | 3:121932624_A/G | 0.24 | 0.20 | 1.01 |
| CaSR | 3:121932701_G/A | 0.17 | 1.00 | 0.99 |
| CaSR | 3:121932854_G/A | 0.24 | 0.20 | 1.01 |
| CaSR | 3:121933066_A/G | 0.24 | 0.20 | 1.01 |
| CaSR | 3:121933505_A/G | 0.24 | 0.20 | 1.01 |
| CaSR | 3:121933635_C/T | 0.24 | 0.20 | 1.01 |
| CaSR | 3:121933833_A/G | 0.24 | 0.19 | 1.01 |
| CaSR | 3:121934161_A/G | 0.17 | 1.00 | 0.99 |
| CaSR | 3:121934177_G/A | 0.27 | 0.13 | 0.99 |
| CaSR | 3:121934360_G/A | 0.24 | 0.19 | 1.01 |
| CaSR | 3:121935408_T/G | 0.49 | 0.96 | 0.99 |
| CaSR | 3:121935826_G/A | 0.24 | 0.19 | 1.01 |
| CaSR | 3:121935846_C/T | 0.27 | 0.39 | 0.98 |
| CaSR | 3:121936101_A/G | 0.24 | 0.19 | 1.01 |
| CaSR | 3:121936107_G/A | 0.24 | 0.19 | 1.01 |
| CaSR | 3:121936200_G/A | 0.24 | 0.19 | 1.01 |
| CaSR | 3:121936267_A/G | 0.24 | 0.19 | 1.01 |
| CaSR | 3:121936278_T/C | 0.24 | 0.19 | 1.01 |
| CaSR | 3:121936323_G/A | 0.27 | 0.39 | 0.98 |
| CaSR | 3:121936370_A/G | 0.27 | 0.38 | 0.98 |
| CaSR | 3:121936453_G/A | 0.07 | 0.91 | 0.90 |
| CaSR | 3:121936532_A/G | 0.49 | 0.91 | 0.99 |
| CaSR | 3:121936773_G/A | 0.17 | 0.91 | 0.99 |
| CaSR | 3:121937353_C/T | 0.29 | 0.32 | 1.00 |
| CaSR | 3:121937943_G/A | 0.15 | 0.86 | 0.99 |
| CaSR | 3:121937961_G/C | 0.15 | 0.88 | 0.99 |
| CaSR | 3:121938454_T/C | 0.07 | 0.31 | 1.01 |
| CaSR | 3:121938744_G/A | 0.07 | 0.31 | 1.01 |
| CaSR | 3:121938903_G/A | 0.25 | 0.35 | 1.01 |
| CaSR | 3:121939414_A/G | 0.17 | 0.66 | 0.99 |
| CaSR | 3:121939743_C/T | 0.12 | 0.88 | 0.96 |
| CaSR | 3:121939770_G/C | 0.29 | 0.44 | 1.00 |
| CaSR | 3:121939975_G/A | 0.25 | 0.35 | 1.01 |
| CaSR | 3:121940165_A/T | 0.07 | 0.31 | 1.01 |
| CaSR | 3:121940209_G/A | 0.29 | 0.43 | 1.00 |
| CaSR | 3:121941456_C/T | 0.25 | 0.35 | 1.01 |
| CaSR | 3:121941750_A/G | 0.29 | 0.43 | 1.00 |
| CaSR | 3:121942302_T/G | 0.25 | 0.35 | 1.01 |
| CaSR | 3:121942812_A/G | 0.24 | 0.34 | 1.01 |
| CaSR | 3:121943859_A/G | 0.25 | 0.34 | 1.01 |
| CaSR | 3:121944349_C/A | 0.25 | 0.34 | 1.01 |
| CaSR | 3:121944811_A/G | 0.17 | 0.68 | 0.99 |
| CaSR | 3:121944857_A/G | 0.17 | 0.68 | 0.99 |
| CaSR | 3:121945164_T/C | 0.25 | 0.34 | 1.01 |
| CaSR | 3:121945470_T/C | 0.25 | 0.36 | 1.00 |
| CaSR | 3:121945805_G/A | 0.07 | 0.31 | 1.01 |
| CaSR | 3:121945838_A/G | 0.25 | 0.36 | 1.00 |
| CaSR | 3:121946099_A/G | 0.25 | 0.36 | 1.00 |
| CaSR | 3:121946277_C/T | 0.17 | 0.68 | 0.99 |
| CaSR | 3:121946297_C/A | 0.17 | 0.68 | 0.99 |
| CaSR | 3:121946355_T/C | 0.25 | 0.34 | 1.01 |
| CaSR | 3:121946376_T/C | 0.25 | 0.34 | 1.01 |
| CaSR | 3:121946738_A/T | 0.25 | 0.34 | 1.01 |
| CaSR | 3:121946765_A/C | 0.25 | 0.37 | 1.00 |
| CaSR | 3:121946769_C/A | 0.17 | 0.66 | 0.99 |
| CaSR | 3:121947301_G/A | 0.25 | 0.36 | 1.01 |
| CaSR | 3:121947525_T/G | 0.25 | 0.36 | 1.01 |
| CaSR | 3:121948114_G/A | 0.29 | 0.48 | 1.00 |
| CaSR | 3:121948645_G/A | 0.17 | 0.64 | 0.99 |
| CaSR | 3:121948686_G/A | 0.13 | 0.89 | 0.98 |
| CaSR | 3:121949292_G/A | 0.29 | 0.49 | 1.00 |
| CaSR | 3:121949401_C/T | 0.23 | 0.95 | 0.97 |
| CaSR | 3:121949745_C/A | 0.17 | 0.64 | 0.99 |
| CaSR | 3:121949970_A/G | 0.25 | 0.36 | 1.01 |
| CaSR | 3:121950065_T/C | 0.25 | 0.38 | 1.00 |
| CaSR | 3:121950068_C/G | 0.25 | 0.38 | 1.00 |
| CaSR | 3:121950120_G/C | 0.29 | 0.48 | 1.00 |
| CaSR | 3:121950136_C/T | 0.17 | 0.64 | 0.99 |
| CaSR | 3:121951055_G/A | 0.07 | 0.31 | 1.01 |
| CaSR | 3:121951483_G/A | 0.07 | 0.31 | 1.02 |
| CaSR | 3:121952452_G/C | 0.17 | 0.66 | 0.99 |
| CaSR | 3:121952945_A/G | 0.17 | 0.64 | 0.99 |
| CaSR | 3:121953794_C/T | 0.17 | 0.64 | 0.99 |
| CaSR | 3:121954077_G/T | 0.11 | 0.88 | 0.99 |
| CaSR | 3:121954304_A/T | 0.30 | 0.25 | 1.00 |
| CaSR | 3:121954311_C/T | 0.17 | 0.64 | 0.99 |
| CaSR | 3:121954680_A/G | 0.11 | 0.79 | 0.99 |
| CaSR | 3:121956409_G/A | 0.17 | 0.63 | 0.99 |
| CaSR | 3:121956953_G/A | 0.13 | 0.89 | 1.00 |
| CaSR | 3:121958171_C/T | 0.06 | 0.89 | 0.99 |
| CaSR | 3:121958475_G/A | 0.11 | 0.94 | 0.94 |
| CaSR | 3:121958850_C/T | 0.17 | 0.61 | 0.99 |
| CaSR | 3:121961137_T/G | 0.17 | 0.61 | 0.99 |
| CaSR | 3:121961461_G/T | 0.07 | 0.31 | 1.02 |
| CaSR | 3:121961573_T/G | 0.13 | 0.97 | 1.01 |
| CaSR | 3:121961685_G/A | 0.17 | 0.61 | 0.99 |
| CaSR | 3:121961880_T/C | 0.13 | 0.95 | 1.01 |
| CaSR | 3:121961931_C/T | 0.13 | 0.95 | 1.01 |
| CaSR | 3:121962006_C/T | 0.06 | 0.72 | 1.00 |
| CaSR | 3:121962246_G/A | 0.30 | 0.27 | 1.00 |
| CaSR | 3:121962478_T/C | 0.42 | 0.65 | 0.99 |
| CaSR | 3:121962963_G/A | 0.30 | 0.27 | 1.01 |
| CaSR | 3:121963025_A/T | 0.13 | 1.00 | 1.01 |
| CaSR | 3:121963285_T/C | 0.30 | 0.27 | 1.01 |
| CaSR | 3:121963858_G/A | 0.13 | 0.87 | 1.01 |
| CaSR | 3:121963877_T/C | 0.13 | 0.87 | 1.01 |
| CaSR | 3:121964084_A/G | 0.17 | 0.63 | 0.99 |
| CaSR | 3:121964262_T/C | 0.30 | 0.27 | 1.01 |
| CaSR | 3:121964374_A/C | 0.13 | 0.87 | 1.01 |
| CaSR | 3:121964798_G/T | 0.17 | 0.63 | 0.99 |
| CaSR | 3:121965199_T/G | 0.13 | 0.97 | 1.01 |
| CaSR | 3:121965246_A/T | 0.30 | 0.24 | 1.01 |
| CaSR | 3:121965400_C/G | 0.17 | 0.63 | 0.99 |
| CaSR | 3:121965745_G/A | 0.07 | 0.31 | 1.01 |
| CaSR | 3:121965791_A/G | 0.06 | 0.72 | 1.00 |
| CaSR | 3:121966156_G/A | 0.13 | 0.89 | 1.01 |
| CaSR | 3:121966233_G/C | 0.13 | 0.92 | 1.01 |
| CaSR | 3:121966240_T/C | 0.17 | 0.61 | 0.99 |
| CaSR | 3:121966370_C/T | 0.17 | 0.63 | 0.99 |
| CaSR | 3:121966638_C/A | 0.30 | 0.24 | 1.00 |
| CaSR | 3:121966942_C/T | 0.17 | 0.63 | 0.99 |
| CaSR | 3:121966952_C/T | 0.24 | 0.54 | 0.97 |
| CaSR | 3:121967297_G/A | 0.13 | 0.92 | 1.01 |
| CaSR | 3:121968090_T/C | 0.30 | 0.24 | 1.00 |
| CaSR | 3:121968267_C/T | 0.06 | 0.72 | 1.00 |
| CaSR | 3:121968552_C/T | 0.13 | 0.92 | 1.01 |
| CaSR | 3:121968847_T/G | 0.30 | 0.24 | 1.00 |
| CaSR | 3:121968898_C/A | 0.17 | 0.61 | 0.99 |
| CaSR | 3:121968905_G/T | 0.17 | 0.57 | 0.99 |
| CaSR | 3:121969712_G/A | 0.13 | 0.92 | 1.01 |
| CaSR | 3:121969937_C/T | 0.13 | 0.97 | 1.00 |
| CaSR | 3:121970020_G/A | 0.17 | 0.63 | 0.99 |
| CaSR | 3:121970131_A/G | 0.30 | 0.25 | 1.00 |
| CaSR | 3:121970231_A/G | 0.17 | 0.70 | 0.99 |
| CaSR | 3:121970301_G/A | 0.30 | 0.24 | 1.00 |
| CaSR | 3:121970386_A/G | 0.30 | 0.24 | 1.00 |
| CaSR | 3:121970949_G/C | 0.11 | 0.91 | 0.95 |
| CaSR | 3:121971011_T/C | 0.17 | 0.77 | 0.98 |
| CaSR | 3:121971070_T/C | 0.13 | 0.95 | 1.00 |
| CaSR | 3:121971438_A/G | 0.17 | 0.75 | 0.98 |
| CaSR | 3:121971512_G/T | 0.07 | 0.31 | 1.00 |
| CaSR | 3:121971802_G/A | 0.30 | 0.20 | 0.99 |
| CaSR | 3:121972370_G/A | 0.16 | 0.91 | 0.96 |
| CaSR | 3:121972635_T/C | 0.16 | 0.65 | 0.96 |
| CaSR | 3:121975327_C/A | 0.42 | 0.21 | 0.85 |
| CaSR | 3:121975353_T/C | 0.12 | 0.77 | 0.91 |
| CaSR | 3:121975485_G/A | 0.13 | 0.84 | 0.98 |
| CaSR | 3:121975596_C/G | 0.08 | 0.26 | 1.01 |
| CaSR | 3:121976494_C/T | 0.49 | 0.95 | 0.99 |
| CaSR | 3:121976547_A/G | 0.49 | 0.96 | 0.99 |
| CaSR | 3:121976926_G/A | 0.49 | 0.95 | 0.99 |
| CaSR | 3:121977125_A/C | 0.18 | 0.27 | 1.00 |
| CaSR | 3:121977282_G/T | 0.33 | 0.58 | 1.00 |
| CaSR | 3:121978073_G/T | 0.33 | 0.61 | 1.00 |
| CaSR | 3:121978343_T/G | 0.14 | 0.41 | 0.99 |
| CaSR | 3:121979229_G/A | 0.14 | 0.41 | 0.99 |
| CaSR | 3:121979690_G/A | 0.27 | 0.43 | 0.99 |
| CaSR | 3:121979704_G/A | 0.36 | 0.96 | 1.00 |
| CaSR | 3:121980186_G/T | 0.11 | 0.94 | 0.98 |
| CaSR | 3:121980242_T/C | 0.32 | 1.00 | 1.00 |
| CaSR | 3:121980284_C/T | 0.33 | 0.61 | 1.00 |
| CaSR | 3:121981609_G/A | 0.14 | 0.40 | 0.99 |
| CaSR | 3:121981619_T/G | 0.35 | 0.93 | 1.00 |
| CaSR | 3:121981836_G/A | 0.35 | 0.95 | 1.00 |
| CaSR | 3:121983607_C/T | 0.14 | 0.44 | 0.99 |
| CaSR | 3:121983805_C/T | 0.35 | 0.97 | 1.00 |
| CaSR | 3:121984021_G/A | 0.13 | 0.74 | 1.00 |
| CaSR | 3:121984044_A/G | 0.11 | 0.97 | 0.99 |
| CaSR | 3:121984792_G/A | 0.14 | 0.43 | 1.00 |
| CaSR | 3:121984850_C/A | 0.11 | 0.97 | 0.99 |
| CaSR | 3:121985345_T/C | 0.32 | 0.86 | 1.01 |
| CaSR | 3:121985358_A/C | 0.32 | 0.86 | 1.01 |
| CaSR | 3:121985608_T/C | 0.32 | 0.89 | 1.01 |
| CaSR | 3:121985666_G/A | 0.12 | 0.83 | 0.92 |
| CaSR | 3:121985882_T/G | 0.35 | 0.99 | 1.00 |
| CaSR | 3:121987328_G/C | 0.14 | 0.72 | 1.00 |
| CaSR | 3:121987587_G/C | 0.13 | 0.79 | 0.99 |
| CaSR | 3:121987619_A/G | 0.35 | 0.97 | 1.00 |
| CaSR | 3:121987647_T/A | 0.32 | 0.97 | 1.01 |
| CaSR | 3:121987932_T/C | 0.11 | 0.94 | 0.99 |
| CaSR | 3:121988034_A/C | 0.32 | 0.99 | 1.01 |
| CaSR | 3:121988120_A/G | 0.13 | 0.74 | 1.00 |
| CaSR | 3:121988355_G/A | 0.32 | 0.99 | 1.01 |
| CaSR | 3:121988505_T/C | 0.13 | 0.74 | 1.00 |
| CaSR | 3:121988738_G/A | 0.13 | 0.74 | 1.00 |
| CaSR | 3:121988851_G/C | 0.14 | 0.43 | 1.00 |
| CaSR | 3:121990228_T/A | 0.35 | 0.95 | 1.00 |
| CaSR | 3:121990257_C/T | 0.33 | 0.51 | 1.01 |
| CaSR | 3:121990800_T/C | 0.14 | 0.43 | 1.00 |
| CaSR | 3:121991083_G/A | 0.15 | 0.44 | 0.99 |
| CaSR | 3:121991139_C/A | 0.35 | 0.92 | 1.00 |
| CaSR | 3:121991213_C/T | 0.35 | 0.91 | 1.00 |
| CaSR | 3:121992825_C/T | 0.08 | 0.20 | 1.03 |
| CaSR | 3:121993247_A/G | 0.15 | 0.44 | 0.99 |
| CaSR | 3:121993432_G/A | 0.13 | 0.79 | 0.99 |
| CaSR | 3:121993474_C/T | 0.46 | 0.13 | 0.99 |
| CaSR | 3:121993600_A/G | 0.33 | 0.49 | 1.01 |
| CaSR | 3:121993641_A/C | 0.13 | 0.74 | 1.00 |
| CaSR | 3:121993837_G/A | 0.11 | 0.90 | 0.99 |
| CaSR | 3:121994941_G/A | 0.08 | 0.20 | 1.03 |
| CaSR | 3:121995415_C/T | 0.08 | 0.20 | 1.03 |
| CaSR | 3:121996083_G/A | 0.13 | 0.74 | 1.00 |
| CaSR | 3:121996235_G/A | 0.05 | 0.15 | 0.96 |
| CaSR | 3:121996810_T/A | 0.08 | 0.18 | 1.03 |
| CaSR | 3:121996816_T/C | 0.32 | 0.99 | 1.01 |
| CaSR | 3:121996883_G/A | 0.32 | 1.00 | 1.01 |
| CaSR | 3:121997268_T/C | 0.08 | 0.18 | 1.03 |
| CaSR | 3:121997388_G/A | 0.35 | 0.97 | 1.00 |
| CaSR | 3:121999059_G/A | 0.08 | 0.22 | 1.03 |
| CaSR | 3:121999155_C/T | 0.11 | 0.90 | 0.99 |
| CaSR | 3:121999651_G/C | 0.14 | 0.43 | 1.00 |
| CaSR | 3:122000134_C/T | 0.35 | 1.00 | 1.00 |
| CaSR | 3:122000667_C/T | 0.14 | 0.69 | 1.00 |
| CaSR | 3:122000871_C/T | 0.32 | 0.97 | 1.01 |
| CaSR | 3:122001099_T/C | 0.13 | 0.77 | 1.00 |
| CaSR | 3:122001246_C/T | 0.32 | 0.97 | 1.01 |
| CaSR | 3:122002178_T/C | 0.14 | 0.46 | 1.00 |
| CaSR | 3:122003757_G/T | 0.15 | 0.50 | 1.00 |
| CaSR | 3:122003769_A/G | 0.07 | 0.25 | 1.03 |
| CaSR | 3:122005053_A/G | 0.35 | 0.88 | 1.00 |
| CaSR | 3:122005131_C/T | 0.14 | 0.58 | 1.00 |
| CaSR | 3:122005236_T/C | 0.32 | 0.86 | 1.02 |
| CaSR | 3:122005273_A/G | 0.10 | 0.65 | 0.99 |

***Table S4:*** *Associations between genetic variants in the Calcium Sensing Receptor (CaSR) and all-cause mortality in colorectal cancer survivors*

|  |  |  | Crude | |  | |  | | Adjusted^1^ | | | | | | | | | |  |
| --- | --- | --- | --- | --- | --- | --- | --- | --- | --- | --- | --- | --- | --- | --- | --- | --- | --- | --- | --- |
|  | **Alleles** | **N/events** | **HR** | | **95%CI** | | **P-value^2^** | | **HR** | | **95%CI** | | **P-value^2^** | | **MAF** | |  | |  |
| 3:121932422_C/T | CC | 6430/2226 | 1.00 | |  | |  | | 1 | |  | |  | | 0.36 | |  | |  |
| rs62269066^a^ | CT | 7239/2480 | 0.98 | | 0.93-1.04 | | 0.5477 | | 0.96 | | 0.91-1.02 | | 0.2189 | |  | |  | |  |
|  | TT | 2065/650 | 0.87 | | 0.79-0.94 | | 0.0012 | | 0.86 | | 0.79-0.94 | | 0.0007 | |  | |  | |  |
|  | Additive model | 15734/5356 | 0.95 | | 0.91-0.98 | | 0.0054 | | 0.94 | | 0.90-0.98 | | 0.0016 | |  | |  | |  |
| 3:121913708_C/T | CC | 6476/2236 | 1 | |  | |  | | 1 | |  | |  | | 0.36 | |  | |  |
| rs17282015^a^ | CT | 7221/2480 | 0.99 | | 0.94-1.05 | | 0.738 | | 0.97 | | 0.92-1.03 | | 0.373 | |  | |  | |  |
|  | TT | 2037/640 | 0.87 | | 0.79-0.95 | | 0.0016 | | 0.86 | | 0.79-0.94 | | 0.001 | |  | |  | |  |
|  | Additive model | 15734/5356 | 0.95 | | 0.91-0.99 | | 0.0091 | | 0.94 | | 0.91-0.98 | | 0.0034 | |  | |  | |  |
| 3:121917298_T/C | TT | 6475/2235 | 1 | |  | |  | | 1 | |  | |  | | 0.36 | |  | |  |
| rs11708053^a^ | TC | 7222/2480 | 0.99 | | 0.94-1.05 | | 0.7308 | | 0.97 | | 0.92-1.03 | | 0.3657 | |  | |  | |  |
|  | CC | 2037/641 | 0.87 | | 0.80-0.95 | | 0.0018 | | 0.86 | | 0.79-0.94 | | 0.0012 | |  | |  | |  |
|  | Additive model | 15734/5356 | 0.95 | | 0.91-0.99 | | 0.0099 | | 0.94 | | 0.91-0.98 | | 0.0037 | |  | |  | |  |
| 3:121917387_A/G | AA | 6476/2235 | 1 | |  | |  | | 1 | |  | |  | | 0.36 | |  | |  |
| rs11711698^a^ | AG | 7221/2480 | 0.99 | | 0.94-1.05 | | 0.7414 | | 0.97 | | 0.92-1.03 | | 0.3734 | |  | |  | |  |
|  | GG | 2037/641 | 0.87 | | 0.80-0.95 | | 0.0019 | | 0.87 | | 0.79-0.94 | | 0.0012 | |  | |  | |  |
|  | Additive model | 15734/5356 | 0.95 | | 0.91-0.99 | | 0.0102 | | 0.94 | | 0.91-0.98 | | 0.0038 | |  | |  | |  |
| 3:121958171_C/T | CC | 13976/4754 | 1 | |  | |  | | 1 | |  | |  | | 0.06 | |  | |  |
| rs11918758 | CT | 1708/578 | 0.99 | | 0.90-1.08 | | 0.7596 | | 1 | | 0.91-1.09 | | 0.9343 | |  | |  | |  |
|  | TT | 50/24 | 1.61 | | 1.08-2.40 | | 0.0205 | | 1.66 | | 1.11-2.47 | | 0.01387 | |  | |  | |  |
|  | Additive model | 15734/5356 | 1.02 | | 0.94-1.10 | | 0.6724 | | 1.03 | | 0.95-1.12 | | 0.5028 | |  | |  | |  |
| 3:121988851_G/C | GG | 11636/4037 | 1 | |  | |  | | 1 | |  | |  | | 0.14 | |  | |  |
| rs6783855^b^ | GC | 3769/1221 | 0.92 | | 0.86-0.98 | | 0.0091 | | 0.91 | | 0.86-0.97 | | 0.0057 | |  | |  | |  |
|  | CC | 329/98 | 0.88 | | 0.72-1.07 | | 0.1922 | | 0.89 | | 0.73-1.09 | | 0.2505 | |  | |  | |  |
|  | Additive model | 15734/5356 | 0.92 | | 0.87-0.98 | | 0.0049 | | 0.92 | | 0.87-0.97 | | 0.0041 | |  | |  | |  |
| 3:121990800_T/C | TT | 11636/4037 | | 1 | |  | |  | | 1 | |  | |  | | 0.14 | |  | |
| rs1604446^b^ | TC | 3769/1221 | | 0.92 | | 0.86-0.98 | | 0.0091 | | 0.91 | | 0.86-0.97 | | 0.0057 | |  | |  | |
|  | CC | 329/98 | | 0.88 | | 0.72-1.07 | | 0.1922 | | 0.89 | | 0.73-1.09 | | 0.2505 | |  | |  | |
|  | Additive model | 15734/5356 | | 0.92 | | 0.87-0.98 | | 0.0049 | | 0.92 | | 0.87-0.97 | | 0.0041 | |  | |  | |
| 3:121983607_C/T | CC | 11655/4041 | | 1 | |  | |  | | 1 | |  | |  | | 0.14 | |  | |
| rs2036399^b^ | CT | 3755/1219 | | 0.92 | | 0.86-0.98 | | 0.0149 | | 0.92 | | 0.86-0.98 | | 0.0073 | |  | |  | |
|  | TT | 324/96 | | 0.88 | | 0.72-1.07 | | 0.224 | | 0.88 | | 0.72-1.08 | | 0.2279 | |  | |  | |
|  | Additive model | 15734/5356 | | 0.92 | | 0.87-0.98 | | 0.0056 | | 0.92 | | 0.87-0.98 | | 0.0047 | |  | |  | |
| 3:122002178_T/C | TT | 11629/4033 | | 1 | |  | |  | | 1 | |  | |  | | 0.14 | |  | |
| rs2134224^b^ | TC | 3776/1225 | | 0.92 | | 0.86-0.98 | | 0.0112 | | 0.92 | | 0.86-0.98 | | 0.0071 | |  | |  | |
|  | CC | 329/98 | | 0.88 | | 0.72-1.07 | | 0.194 | | 0.89 | | 0.73-1.09 | | 0.2527 | |  | |  | |
|  | Additive model | 15734/5356 | | 0.92 | | 0.87-0.98 | | 0.0062 | | 0.92 | | 0.87-0.98 | | 0.005 | |  | |  | |
| 3:121978343_T/G | TT | 11645/4037 | | 1 | |  | |  | | 1 | |  | |  | | 0.14 | |  | |
| rs62269091^b^ | TG | 3762/1223 | | 0.92 | | 0.86-0.98 | | 0.0124 | | 0.92 | | 0.86-0.98 | | 0.0078 | |  | |  | |
|  | GG | 327/96 | | 0.86 | | 0.70-1.05 | | 0.1305 | | 0.87 | | 0.71-1.07 | | 0.1804 | |  | |  | |
|  | Additive model | 15734/5356 | | 0.92 | | 0.87-0.98 | | 0.0046 | | 0.92 | | 0.87-0.97 | | 0.004 | |  | |  | |

1: Adjusted for age at diagnosis, sex, cohort and the first three principal components of genetic ancestry

2: The estimated effective number of independent tests among 325 SNPS was 35 based on the simple M approach. Therefore, p-values <0.001 were considered statistically significant (n=35; 0.05/35=0.001).

^a, b^ Correlated SNPs (r^2^>0.6)

***Table S5:*** *Associations between genetic variance in the Calcium Sensing Receptor (CaSR) and colorectal cancer specific mortality*

|  |  |  | Crude |  |  | Adjusted^1^ | | | | |
| --- | --- | --- | --- | --- | --- | --- | --- | --- | --- | --- |
|  | **Alleles** | **N/events** | **HR** | **95%CI** | **P-value^2^** | **HR** | **95%CI** | **P-value^2^** | **MAF** |  |
| 3:122005273_A/G | AA | 12645/2852 | 1 |  |  | 1 |  |  | 0.1 |  |
| rs34042920^a^ | AG | 2910/597 | 0.9 | 0.82-0.98 | 0.018 | 0.89 | 0.82-0.97 | 0.01 |  |  |
|  | GG | 179/44 | 1.1 | 0.82-1.48 | 0.5247 | 1.13 | 0.84-1.52 | 0.4258 |  |  |
|  | Additive model | 157343/3493 | 0.93 | 0.86-1.01 | 0.0746 | 0.93 | 0.86-1.00 | 0.0573 |  |  |
| 3:121988851_G/C | GG | 11636/2635 | 1 |  |  | 1 |  |  | 0.14 |  |
| rs6783855^a^ | GC | 3769/791 | 0.91 | 0.84-0.99 | 0.0253 | 0.91 | 0.84-0.98 | 0.0151 |  |  |
|  | CC | 329/67 | 0.91 | 0.71-1.16 | 0.436 | 0.92 | 0.72-1.17 | 0.4901 |  |  |
|  | Additive model | 157343/3493 | 0.92 | 0.86-0.99 | 0.0251 | 0.92 | 0.86-0.99 | 0.0184 |  |  |
| 3:121990800_T/C | TT | 11636/2635 | 1 |  |  | 1 |  |  | 0.14 |  |
| rs1604446^a^ | TC | 3769/791 | 0.91 | 0.84-0.99 | 0.0253 | 0.91 | 0.84-0.98 | 0.0151 |  |  |
|  | CC | 329/67 | 0.91 | 0.71-1.16 | 0.436 | 0.92 | 0.72-1.17 | 0.4901 |  |  |
|  | Additive model | 157343/3493 | 0.92 | 0.86-0.99 | 0.0252 | 0.92 | 0.86-0.99 | 0.0184 |  |  |
| 3:121983607_C/T | CC | 11655/2639 | 1 |  |  | 1 |  |  | 0.14 |  |
| rs2036399^a^ | TC | 3755/788 | 0.91 | 0.84-0.99 | 0.0264 | 0.91 | 0.84-0.98 | 0.0165 |  |  |
|  | TT | 324/66 | 0.91 | 0.71-1.16 | 0.4237 | 0.92 | 0.72-1.17 | 0.4928 |  |  |
|  | Additive model | 157343/3493 | 0.92 | 0.86-0.99 | 0.0255 | 0.92 | 0.86-0.99 | 0.0191 |  |  |
| 3:122002178_T/C | TT | 11629/2633 | 1 |  |  | 1 |  |  | 0.14 |  |
| rs2134224^a^ | TC | 3776/793 | 0.91 | 0.84-0.99 | 0.02718 | 0.91 | 0.84-0.98 | 0.0165 |  |  |
|  | CC | 329/67 | 0.91 | 0.71-1.16 | 0.4372 | 0.92 | 0.72-1.17 | 0.4916 |  |  |
|  | Additive model | 157343/3493 | 0.92 | 0.86-0.99 | 0.0268 | 0.92 | 0.86-0.99 | 0.0199 |  |  |
| 3:121999651_G/C | GG | 11636/2634 | 1 |  |  | 1 |  |  | 0.14 |  |
| rs3804595^a^ | GC | 3769/792 | 0.92 | 0.85-0.99 | 0.0291 | 0.91 | 0.84-0.98 | 0.0175 |  |  |
|  | CC | 329/67 | 0.91 | 0.71-1.16 | 0.4385 | 0.92 | 0.72-1.17 | 0.4928 |  |  |
|  | Additive model | 157343/3493 | 0.93 | 0.86-0.99 | 0.0284 | 0.92 | 0.86-0.99 | 0.0209 |  |  |
| 3:121978343_T/G | TT | 11645/2636 | 1 |  |  | 1 |  |  | 0.14 |  |
| rs62269091^a^ | TG | 3762/791 | 0.91 | 0.85-0.99 | 0.0284 | 0.91 | 0.84-0.98 | 0.0175 |  |  |
|  | GG | 327/66 | 0.89 | 0.70-1.14 | 0.3716 | 0.91 | 0.71-1.16 | 0.4931 |  |  |
|  | Additive model | 157343/3493 | 0.92 | 0.86-0.99 | 0.0238 | 0.92 | 0.86-0.99 | 0.0182 |  |  |
| 3:121932422_C/T | CC | 6430/1463 | 1 |  |  | 1 |  |  | 0.36 |  |
| rs62269066 | CT | 7239/1602 | 0.97 | 0.90-1.04 | 0.3968 | 0.96 | 0.89-1.03 | 0.2593 |  |  |
|  | TT | 2065/428 | 0.88 | 0.79-0.98 | 0.0216 | 0.88 | 0.79-0.98 | 0.0197 |  |  |
|  | Additive model | 157343/3493 | 0.95 | 0.90-1.00 | 0.0376 | 0.94 | 0.90-0.99 | 0.0221 |  |  |
| 3:121927745_G/A | GG | 4085/920 | 1 |  |  | 1 |  |  | 0.49 |  |
| rs17203502^b^ | GA | 7861/1785 | 1 | 0.93-1.09 | 0.9088 | 1 | 0.92-1.08 | 0.946 |  |  |
|  | AA | 3788/788 | 0.9 | 0.82-0.99 | 0.0335 | 0.9 | 0.81-0.99 | 0.0234 |  |  |
|  | Additive model | 157343/3493 | 0.95 | 0.91-1.00 | 0.0385 | 0.95 | 0.90-0.99 | 0.0269 |  |  |
| 3:121924998_A/G | AA | 4054/917 | 1 |  |  | 1 |  |  | 0.49 |  |
| rs55639113^b^ | AG | 7843/1774 | 1 | 0.92-1.08 | 0.9088 | 0.99 | 0.91-1.07 | 0.7781 |  |  |
|  | GG | 3837/802 | 0.9 | 0.82-0.99 | 0.0342 | 0.9 | 0.82-0.99 | 0.0244 |  |  |
|  | Additive model | 157343/3493 | 0.95 | 0.91-1.00 | 0.0376 | 0.95 | 0.90-0.99 | 0.0267 |  |  |

1: Adjusted for age at diagnosis, sex, cohort and the first three principal components of genetic ancestry

2: The estimated effective number of independent tests among 325 SNPS was 35 based on the simple M approach. Therefore, p-values <0.001 were considered statistically significant (n=35; 0.05/35=0.001).

^a,b^ Correlated SNPs (r^2^>0.6)

***Figure S2****: Forest plots for the association between dietary calcium intake, supplemental calcium intake and total calcium intake in relation to CRC-specific and all-cause mortality in CRC survivors for each included study separately and the weighted merged meta-analysis.*

| All-cause mortality | CRC-specific mortality |
| --- | --- |
| Dietary calcium intake (sex and cohort specific quartiles) |  |
| 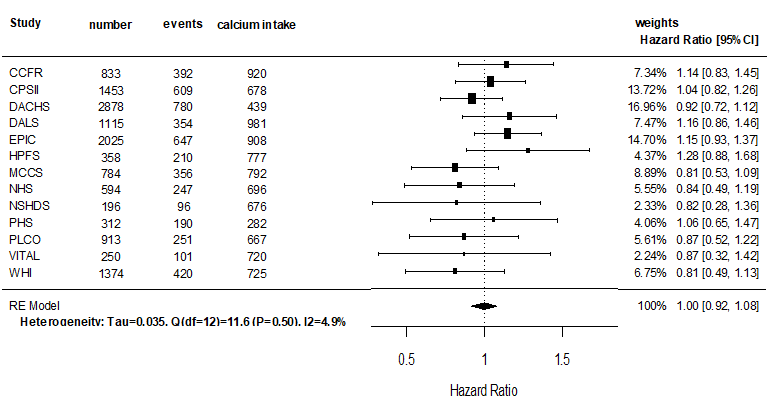 | 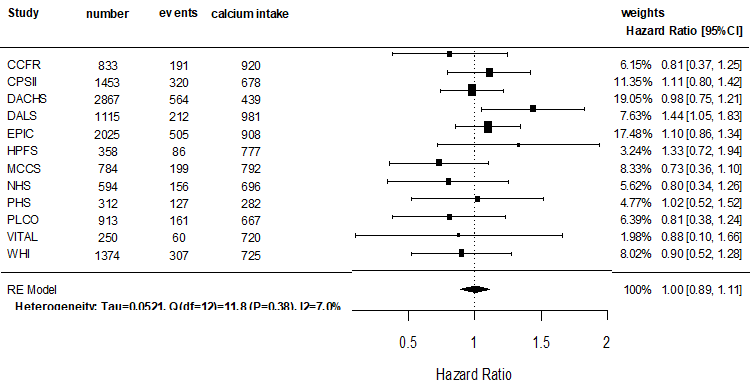 |

| Supplemental calcium intake (≥1 versus < 1 pill) |  |
| --- | --- |
| 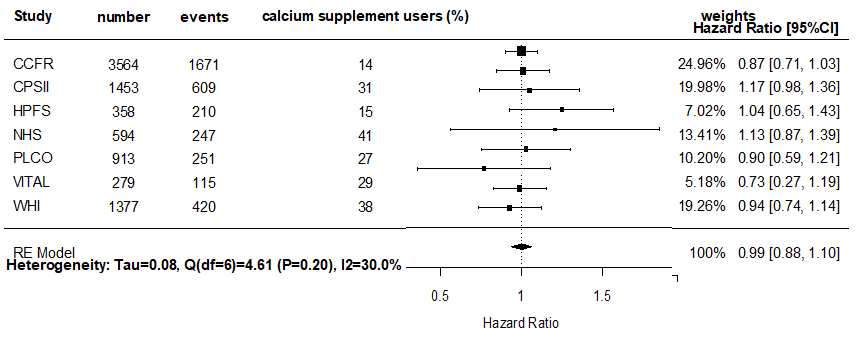 | 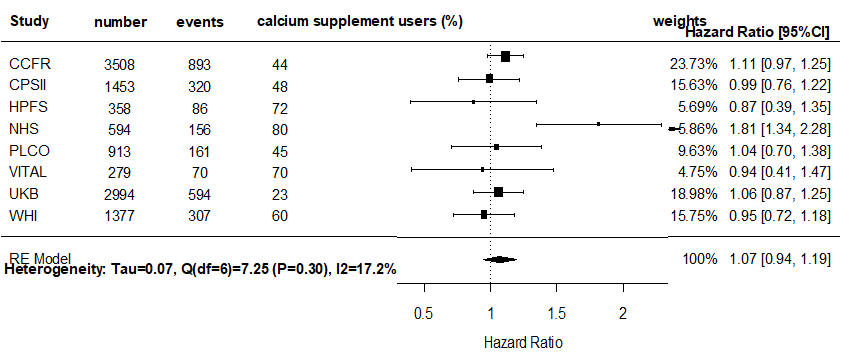 |
| Total calcium intake (sex and cohort specific quartiles) |  |
| 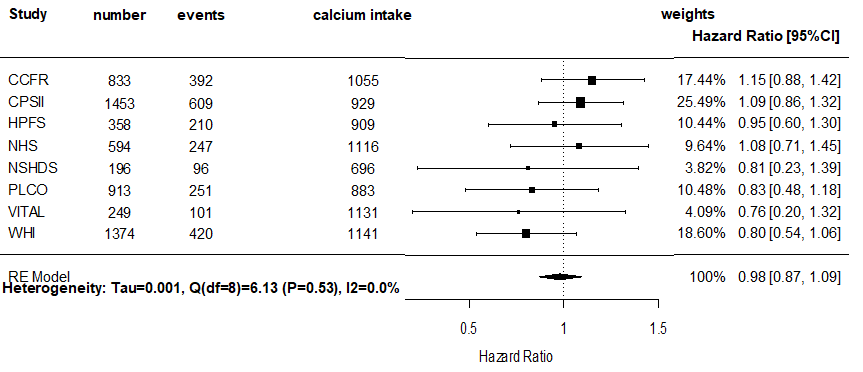 | 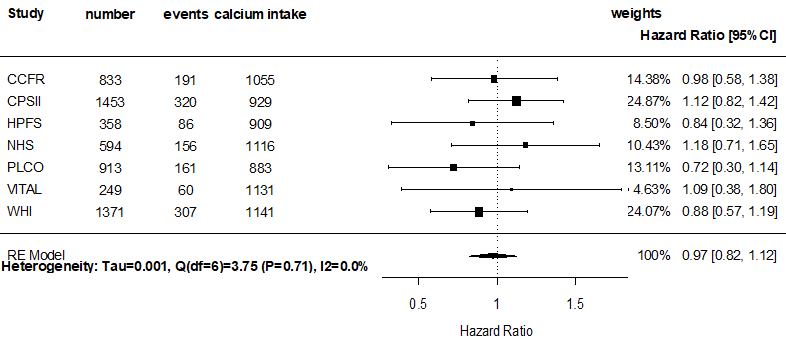 |
